# Supplementary material for: Hydrostratigraphy and hydrogeophysical studies to delineate fresh and saline aquifer boundaries in Lesser Cholistan of Pakistan
Source: PLoS One. 2023 Oct 5;18(10):e0292035. doi: 10.1371/journal.pone.0292035 (PMC10553205; doi:10.1371/journal.pone.0292035)

PLOS ONE

Hydrostratigraphy and Hydrogeophysical studies to delineate fresh and saline aquifer boundaries in Lesser Cholistan of Pakistan

--Manuscript Draft--

**Manuscript Number:**	PONE-D-23-07514
**Article Type:**	Research Article
**Full Title:**	Hydrostratigraphy and Hydrogeophysical studies to delineate fresh and saline aquifer boundaries in Lesser Cholistan of Pakistan
**Short Title:**	Hydrostratigraphy and Hydrogeophysical studies to delineate fresh and saline aquifer boundaries
**Corresponding Author:**	PERVEIZ KHALID, Ph.D.<br>University of the Punjab<br>Lahore, PAKISTAN
**Keywords:**	Electrical resistivity; Saline groundwater; Quaternary sediments; Cholistan Desert
**Abstract:**	The differentiation of saline water and fresh water interfaces is the key objective in ground water exploration and management. Bahawalpur is the twelfth biggest metropolitan of Pakistan situated in south Punjab near to bank of River Sutlej ~~Pakistan~~ and lies at 29°39’55” N Latitude and 73°15’12” E Longitude at an elevation of 521 ft AMSL in the Cholistan area close to the Thar Abandon where a supply of fresh water is challenging because of high salinity values in groundwater. Vertical electrical resistivity and borehole data are used to mark fresh and saline interfaces of groundwater. To achieve the results 230 vertical electric soundings were performed in the study area. A total 3 to 5 geo-electric layers are identified with modeling along with the processing and interpretation of resistivity data. Bore hole data is used to interpret subsurface lithologies and to calibrate the modeled resistivity curves. The electric resistivity data indicates that thick layers of Quaternary sediments are present in the subsurface ~~that is primarily composed of clay, silt, sand, gravels and some kankar~~. Inversion technique is applied to generate 2D and 3D subsurface resistivity maps to delineate fresh and saline water zones. The generated 2D resistivity maps at variable depth above and below water table and formation resistivity maps are successfully utilized to differentiate fresh and saline water zones. The identification of a saline water aquifer within sediments rich in clay was made possible by the observation of very low resistivity measurements in the southern region. Conversely, the detection of relatively high resistivity values, coupled with the presence of sand and gravel deposits in the northern section of the lesser Cholistan area, provided compelling evidence of the existence of fresh groundwater. These findings have significant implications for the management of water resources in the region, as they provide valuable insights into the distribution and availability of groundwater resources for future use.
**Order of Authors:**	PERVEIZ KHALID, Ph.D.
	KASHIF ARIF
**Additional Information:**	
**Question**	**Response**
**Financial Disclosure**<br><br>Enter a financial disclosure statement that describes the sources of funding for the work included in this submission. Review the submission guidelines for detailed requirements. View published research articles from *PLOS ONE* for specific examples.	The author(s) received no specific funding for this work.

Powered by Editorial Manager® and ProduXion Manager® from Aries Systems Corporation

This statement is required for submission and **will appear in the published article** if the submission is accepted. Please make sure it is accurate.<br><br>**Unfunded studies**<br>Enter: *The author(s) received no specific funding for this work.*<br><br>**Funded studies**<br>Enter a statement with the following details:<br>• Initials of the authors who received each award<br>• Grant numbers awarded to each author<br>• The full name of each funder<br>• URL of each funder website<br>• Did the sponsors or funders play any role in the study design, data collection and analysis, decision to publish, or preparation of the manuscript?<br>• **NO** - Include this sentence at the end of your statement: *The funders had no role in study design, data collection and analysis, decision to publish, or preparation of the manuscript.*<br>• **YES** - Specify the role(s) played.<br><br>* typeset	
**Competing Interests**<br><br>Use the instructions below to enter a competing interest statement for this submission. On behalf of all authors, disclose any competing interests that could be perceived to bias this work—acknowledging all financial support and any other relevant financial or non-financial competing interests.<br><br>This statement is required for submission and **will appear in the published article** if the submission is accepted. Please make sure it is accurate and that any funding sources listed in your Funding Information later in the submission form are also declared in your Financial Disclosure statement.<br><br>View published research articles from *PLOS ONE* for specific examples.	The authors have declared that no competing interests exist.

Powered by Editorial Manager® and ProduXion Manager® from Aries Systems Corporation

**NO authors have competing interests**<br><br>Enter: *The authors have declared that no competing interests exist.*<br><br>**Authors with competing interests**<br><br>Enter competing interest details beginning with this statement:<br><br>*I have read the journal's policy and the authors of this manuscript have the following competing interests: [insert competing interests here]*<br><br>* typeset	
**Ethics Statement**<br><br>Enter an ethics statement for this submission. This statement is required if the study involved:<br><br>• Human participants<br>• Human specimens or tissue<br>• Vertebrate animals or cephalopods<br>• Vertebrate embryos or tissues<br>• Field research<br><br>Write "N/A" if the submission does not require an ethics statement.<br><br>General guidance is provided below. Consult the submission guidelines for detailed instructions. **Make sure that all information entered here is included in the Methods section of the manuscript.**	N/A

Powered by Editorial Manager® and ProduXion Manager® from Aries Systems Corporation

**Format for specific study types**<br><br>**Human Subject Research (involving human participants and/or tissue)**<br>• Give the name of the institutional review board or ethics committee that approved the study<br>• Include the approval number and/or a statement indicating approval of this research<br>• Indicate the form of consent obtained (written/oral) or the reason that consent was not obtained (e.g. the data were analyzed anonymously)<br><br>**Animal Research (involving vertebrate animals, embryos or tissues)**<br>• Provide the name of the Institutional Animal Care and Use Committee (IACUC) or other relevant ethics board that reviewed the study protocol, and indicate whether they approved this research or granted a formal waiver of ethical approval<br>• Include an approval number if one was obtained<br>• If the study involved *non-human primates*, add *additional details* about animal welfare and steps taken to ameliorate suffering<br>• If anesthesia, euthanasia, or any kind of animal sacrifice is part of the study, include briefly which substances and/or methods were applied<br><br>**Field Research**<br><br>Include the following details if this study involves the collection of plant, animal, or other materials from a natural setting:<br>• Field permit number<br>• Name of the institution or relevant body that granted permission	
**Data Availability**<br><br>Authors are required to make all data underlying the findings described fully available, without restriction, and from the time of publication. PLOS allows rare exceptions to address legal and ethical concerns. See the PLOS Data Policy and FAQ for detailed information.	No - some restrictions will apply

A Data Availability Statement describing where the data can be found is required at submission. Your answers to this question constitute the Data Availability Statement and **will be published in the article**, if accepted.<br><br>**Important:** Stating 'data available on request from the author' is not sufficient. If your data are only available upon request, select 'No' for the first question and explain your exceptional situation in the text box.<br><br>Do the authors confirm that all data underlying the findings described in their manuscript are fully available without restriction?	
**Describe where the data may be found in full sentences. If you are copying our sample text, replace any instances of XXX with the appropriate details.**<br><br>• If the data are **held or will be held in a public repository**, include URLs, accession numbers or DOIs. If this information will only be available after acceptance, indicate this by ticking the box below. For example: *All XXX files are available from the XXX database (accession number(s) XXX, XXX.*).<br>• If the data are all contained **within the manuscript and/or Supporting Information files**, enter the following: *All relevant data are within the manuscript and its Supporting Information files.*<br>• If neither of these applies but you are able to provide **details of access elsewhere**, with or without limitations, please do so. For example:<br><br>*Data cannot be shared publicly because of [XXX]. Data are available from the XXX Institutional Data Access / Ethics Committee (contact via XXX) for researchers who meet the criteria for access to confidential data.*<br><br>*The data underlying the results presented in the study are available from (include the name of the third party*	The data will be available on electron request to the corresponding author.

and contact information or URL).<br>• This text is appropriate if the data are owned by a third party and authors do not have permission to share the data.<br><br>* typeset	
Additional data availability information:	Tick here if your circumstances are not covered by the questions above and you need the journal's help to make your data available.

Powered by Editorial Manager® and ProduXion Manager® from Aries Systems Corporation

Manuscript

Click here to access/download;Manuscript;paper 1.docx

Hydrostratigraphy and Hydrogeophysical studies to delineate fresh and saline aquifer boundaries in Lesser Cholistan of Pakistan

Kashif Arif[1], Perveiz Khalid[1]

[1]Institute of Geology, University of the Punjab 54590 Lahore, Pakistan

*Email: perveiz.geo@pu.edu.pk

Abstract

The differentiation of saline water and fresh water interfaces is the key objective in ground water exploration and management. Bahawalpur is the twelfth biggest metropolitan of Pakistan situated in south Punjab near to bank of River Sutlej Pakistan and lies at 29°59’55” N Latitude and 73°15’12” E Longitude at an elevation of 521 ft AMSL in the Cholistan area close to the Thar Abandon where ~~continuous~~ supply of fresh water is challenging because of high salinity values in groundwater. Vertical electrical resistivity and borehole data are used to mark fresh and saline interfaces of groundwater. To achieve the results 230 vertical electric sounding were performed in the study area. A total 3 to 5 geo-electric layers are identified with modeling along with the processing and interpretation of resistivity data. Bore hole data is used to interpret subsurface lithologies and to calibrate the modeled resistivity curves. The electric resistivity data indicates that thick layers of Quaternary sediments is present in the subsurface that is primarily composed of clay, silt, sand, gravels and some kankar. Inversion technique is applied to generate 2D and 3D subsurface resistivity maps to delineate fresh and saline water zones. The generated 2D resistivity maps at variable depth above and below water table and formation resistivity maps are successfully utilized to differentiate fresh and saline water zones. The identification of a saline water aquifer within sediments rich in clay was made possible by the observation of very low resistivity

measurements in the southern region. Conversely, the detection of relatively high resistivity values, coupled with the presence of sand and gravel deposits in the northern section of the lesser Cholistan area, provided compelling evidence of the existence of fresh groundwater. These findings have significant implications for the management of water resources in the region, as they provide valuable insights into the distribution and availability of groundwater resources for future use.

Keywords Electrical resistivity, Saline groundwater, Quaternary sediments, Cholistan

Introduction

The sustainable supply of freshwater is one of the key parameters in urbanization and the socio-economic development of an area (Muhammad et al., 2022). Surface water, which includes streams, canals, lakes, rivers, etc. and groundwater are two primary cheap and easily available sources of freshwater (Hasan, 2017). Due to high solubility of different solutes water is capable to dissolve suspend, soak and adsorb various impurities such as arsenic, sulfides, and chlorides, organic and inorganic etc. (Kosemani and Oyelami, 2017). Pakistan is among those countries that are facing freshwater scarcity. The whole country is situated in arid to the semi-arid regions with low precipitation. The majority of the population relies on groundwater for their domestic, agricultural and industrial needs. The over-abstraction of groundwater is one of the main ~~reason in enhancing~~ salinity in the groundwater whereas the poor dumping strategies of solid waste is the main source of groundwater contamination (Rasool, 2017).

Cholistan desert – a part of Thar desert – covers an area of 25,800 km^2 is the largest desert in the southern Punjab province of Pakistan (Figure 1). This desert is divided into two part[illegible] Lesser Cholistan and Higher Cholistan. Bahawalpur city is the largest city in the Lesser Cholistan while the Higher Cholistan is uninhabited with high sand dunes upto 100 m. Around 16,000 km^2 of the district is comprised of the Cholistan Desert that is covered by dunes of low height (Farooq et al., 2001). The density of dunes increases towards the northwest. The economy of this district mainly based on agriculture thus the continuous supply of freshwater is the backbone in its economy. However, the existing groundwater is highly contaminated by hazardous pollutants. The analysis of groundwater performed by PCRWR from 2002 to 2015 demonstrates that the groundwater on most of the localities in this district is not safe for drinking. In the southern part of the Bahawalpur area, between Fort Abbas and Derawar Fort, there is a zone of reduced salinity. This relatively

low-lying area corresponds to the ancient channel of Ghaggar (Hakra) River. Although the Ghagger (Hakra) channel is now generally dry, floodwaters occasionally flow through it from the east, near Fort Abbas, in periods of uncommonly high rainfall. Available data indicate that the upper part of the aquifer along this channel contains water of about 4,000 ppm TDS. A deep-water sample (~ 410 m depth) collected near to Derawar Fort contained high salt concentration up to 25,400 ppm (Geyh and Ploethner, 2008).

Fig. 1: *Base map of the study area showing VES and Borehole locations.*

The study area is a part of the Punjab Platform that is a subdivision of the Upper Indus Basin. The study area consists of alluvial Aeolian plains (Farooq et al., 2001). The area along the Sutlej River comprised the alluvial plain whereas the rolling dunes cover the Aeolian plain of the Cholistan Desert. The planning and management for better quality water supply are not much efficient in Bahawalpur City (Anwar and Bureste, 2011). The use of hydrogeological investigations with the help of some appropriate geophysical techniques and methods to assess the groundwater potential in confined or unconfined aquifer system in a particular area is very common (Courteaud et al., 1997; Zouhri et al., 2004; Schrott and Sass, 2008; Edmund, 2009; Okoro et al., 2010; Riddell et al., 2010; Muchingami et al., 2012; Utom et al., 2012; Vouillamoz et al., 2012; Farid et al., 2013; Mondal et al., 2013; Farid et al., 2014). The spatial and vertical distribution of different characteristics of aquifer such as facies distribution and hydraulic parameters variations are fundamental for the study of alluvial deposits.

Since the saline water has an enormous amount of dissolved solids (TDS), therefore, modeling and delineation of fresh and saline water boundaries in the subsurface are necessary

before planning of water abstraction and installation of tube wells. The primary objective of this work is to delineate fresh and saline water interfaces and to map the fresh water aquifer boundaries.

Geology and Geomorphology of the Area

Pakistan is situated at the collisional boundary of the Indian and Eurasian Plates, which creates a significant number of intermontane basins and valley systems (Farid et al., 2018). Indus basin is one of the basins produced under the action of local tectonic activities. The sediments generated by the erosion of the surrounding mountains are transported into this basin under the action of sedimentary transport agents. These sediments may vary in size from very coarse alluvial fan gravels and channel deposits with high hydraulic conductivities to very fine lacustrine clays with low hydraulic conductivity and less permeability. Thus, the grain size of the sediments, lithology and geomorphic nature of the deposited sediments are the important agents for the development of clastic fluvial aquifers and their hydraulic characteristics (Fetter, 1994; Bowling et al., 2005). Since the study area is far away from the source of sediments therefore, fine-grained silt/clay to medium to coarse-grained sand are present only.

The borehole data suggests that a 457 m thick cover of alluvium sediments comprising of fine-grained silt and clay to medium to coarse sand of Late Quaternary age is present throughout the area (Shamsi et al., 1965). This cover is of heterogeneous in nature with significant lateral and horizontal variation in lithology (Kemal et al., 1965; Hassan, 1965). Thus, more than one aquifer system may be present in the study area. According to NESPAK report (1980), the alluvium is saturated up to 1,000 m depth. No continuous layer of clay is reported above the aquifer, thus an unconfined type of aquifer may present in the area. However, locally confined or semi-confined aquifer conditions may prevail. The landforms are developed by the action of wind and river and consist of two units: flood plain deposits and aeolian deposits. The width of flood plains varies

from 10 – 20 km in different localities. According to geomorphological classification, the study area consists of level plains, channel infills, basin, and levees as shown in Fig. 2.

Fig. 2: *Digital elevation model shows major geomorphology of the area.*

The sediments deposited in the area are mainly fine to medium grains silt, clay, and sands in the association of Kanker. Since the area is far away from the source of sediments, therefore coarser sediments such as gravels are very rare. The sand plains of Cholistan Desert comprised of some medium-height sandy ridges and inter-dunal hollows of Hakra River. These deposits are composed of fine to medium silty sand and silt with low permeability. These sediments are well rounded, well-sorted, and transported by wind from adjacent arid zones (Fig. 3 & Fig. 4). An old channel of Hakra River forms a freshwater channel (Geyh and Ploethner, 2015). In the 16th century, this river was ceased running (Wilhelmy, 1969) and only rainwater flow in it until mid of the 20th century. This former fluvial environment exhibits a flat, hardpan surface on which dunes irregularly rest up to10 m high. The upper layer of the floodplain comprises fine-grained silt to sandy loam and silty clays of low permeability but good porosity. Thus, rainwater may accumulate in depressions and may stay for a couple of days to several weeks. Since 1960, some portion of this desert has been converted into cultivated land after the construction of canals starting from Sutlej River. The area receives less than 200 mm of rainfall annually.

Fig. 3: *Geological Map of Cholistan Desert.*

Rainwater and the Sutlej River are the main recharge sources in the study area. Due to the presence of less permeable top cover on the surface, rainwater does not penetrate to a deeper extent. The recharge rate can be divided into deep and shallow categories. The palaeohydrological situation can be used to explain different recharge rates for shallow and deep aquifer layers. No reliable temporal information is available in the western part of the Cholistan area. However, pluvial conditions existed between 13 000 and about 4000 years ago in the eastern part of the Cholistan (Singh et al. 1972, 1974; Wasson et al.; 1984). According to literature, two pluvial periods may have existed from 13,000 to 8,000 years and 7,000 to 4,000 years (Geyh and Ploethner, 2015).

A few numbers of studies related to hydrogeophysical investigations and groundwater quality are available in the literature. In 1968, USGS conducted a study on groundwater hydrology of the Punjab Province. According to this study, Most of the area of district Bahawalpur comprises highly saline water with TDS> 5000 ppm except a narrow freshwater corridor along the Sutlej River and an old channel of Hakra River between Fort Abbas to Derawar Fort. Farooq et al. (2001) conducted a study on physio-chemical analysis of shallow water samples collected from 20 – 70 m depth. They concluded that the groundwater is highly mineralized in most of the area of Rahim Yaar Khan District with a high concentration of TDS (27,000 ppm) and high sodium absorption ration ~ 54 mg/l).

Fig. 4*: Boreholes in the study area representing the shallow subsurface geology*.

Methodology

Electrical resistivity has long been used as a method to detect the subsurface hydrogeological and geomorphologic features with considerable success (Stewart et al. 1983; Courteaud et al. 1997; Louis et al., 2002; Zouhri et al., 2004; Riddell et al., 2010). Electrical resistivity in addition to the lithologic makeup of the aquifer depends upon the fluid contents of the aquifer. The methodology involve digitations of existing data and the acquired data set. The acquired data comprise of vertical electrical soundings (VES) up to 300 m depth at sparsely distributed locations for convenience, the VES points were generally collected at distance of 4 to 8 km, mostly along the roads, canals and existing tracks as shown in Fig. 1. A terrameter SAS 4000 is used for VES data acquisition. All VES data were acquired by vertical sounding using Schlumberger array configuration. The Schlumberger array consists of four electric conductors known as electrodes. The two end conductors are current electrodes and the inside two conductors are the potential electrodes. The potential electrodes are installed at the centre of the electrode array with a small separation, typically less than one-fifth of the spacing between the current electrodes ($AB \geq 5MN$). The current electrodes are increased to a greater separation during the survey while the potential electrodes remain in the same position until the observed voltage becomes too small to measure.

The data of around 120 test holes, 20 test wells up to 300 m depth is already available from the Water and Power Development Authority (WAPDA) of Pakistan. More than 230 vertical electrical resistivity sounding (VES) data are acquired in different places of the study area was used for the characterization of various subsurface lithologies. VES data is interpreted by using a combination of curve matching technique and computer iterative modeling to find out number of geo-electric layers in the study area. The analysis of VES curves is difficult task and requires that the measured curve be matched with several model curves and each model curve represent different subsurface resistivity distributions. To select the final model is constrained with the help

of VES data, lithologs, local geological data, and borehole data of study area, we map different depositional zones in the Bahawalpur district to mark the alluvial aquifer systems as well as the salinity of the groundwater. For calibration and verification of VES results with borehole data, some of the VES points are acquired near to the existing boreholes/tubewells. All the VES data points were modeled (Fig.5) by using computer software IP2Win (IPI2WIN-1D computer program. 2000; Zananiri et al., 2006; Sultan et al., 2009; Akhter et al., 2012; Farid et al., 2013; Farid et al., 2014; Muhammad and Khalid, 2017), using information derived from lithology logs, ground water levels, geologic maps and EC maps. 2 D models are generated and plotted against the resistivity values verses depth and consist of several horizontal layers separated according to discrete band of resistivity as shown in Fig. 5a-f. In general calibration between lithology and resistivity is established using available borehole information and resistivity data and is shown in Table 1 and in Fig. 6.

Table 1: *Cutoff values of resistivity for different lithologies used in interpretation of VES data.*

Resistivity Zone	Resistivity Range (Ω-m)	Interpreted Lithology
Very high	Greater than 230	Coarse sand with gravels and kanker
High	Less than 230 to 100	sand with gravels with some interbeded thin layer of silt and clay
Medium	Less than 100 to 40	Medium to coarse sand
Low	Less than 40 to 20	Mixture of sand with some silt and clay

		Silty clay / clayey silt (Above GWT)
Very low	Less than 20	Saline sediments (Below GWT)

The details provided in Table. 1 has been used to analyze all of the VES curves and developing layer models. Each model response curve represents the virtual VES response of horizontally stratified earth layer using a limited number of sedimentary layers (3-5). The base layer in each model extends to undefined depth value.

Fig. 5a-f: *Apparent resistivity data are marked by small circles. Solid black curve represent the apparent resistivity curve. Red curve is the best fitted curve to apparent resistivity data. Solid blue block line is the modeled resistivity (synthetic resistivity). Horizontal axis is the current electrode spacing (AB/2) in meters and vertical axis is the resistivity in ohm meters*.

Fig .6: *Borehole logs and modeled resistivity curves representing the calibration between lithology and resistivity data.*

Result and discussion

Interpretations are made for three to five numbers of geo-electric subsurface layers in the study area. The resistivity and depth/thickness of each layer is distinctive. Electrical resistivity value of each geo-electric layer which can be implemented to characterize the probable underlying lithology, is a function of particle size, porosity, and fluid type. A correlation between the

interpreted underlying lithology and true resistivities is created by simultaneously examining the processed data from 230 VES probes and lithological logs of boreholes (Table 1). Furthermore, the lithological logs of boreholes closest to the respective VES probes are used to calibrate the true resistivities (Fig. 6).

Clay, silt, and sand comprise the alluvial deposits with gravel and pebbles of different depths, can be encountered in boreholes that serve as aquifers. The southern portion of the lesser Cholistan has clay-rich sediments as a result of the intrusion of salt contents, and the findings of the resistivity inversion provide confirmation of this. The interpretation of VES results in this area was challenging due to the relatively low difference of resistivity readings between shallow and deep alluvium. Sand and gravel sediments are more prominent in the north and northwestern part of the study area, where they serve as a good aquifer material for fresh groundwater, and clay rich sediments are more dominant in the south and southeast, where it serves as a saline water aquifer, according to borehole data shown in Fig. 4. According to Fig. 2 digital elevation map of Cholistan, where the elevation decline from northeast to southwest, the streams run in a north to southwest direction. The digital elevation map and the ground water level are interrelated. The ground water level changes across the Lesser Cholistan. In the northeast at well TH-50, the water table is around $\leq$ 12m deep, while in the southwest borehole TH-91, it is about $\leq$ 17m deep. As can be seen in Fig. 8a-h, the electrical resistivity survey findings indicate that the south portion of the Lesser Cholistan has significantly lower resistivity values than the north side. In the south region of the study area, where the depth of penetration in the VES findings is very low, resistivity data results confirm that higher proportions of clay-rich or salty sediments prevent the electric current from flowing at greater depths. The dry sand, gravel, and boulder sediments are linked with high resistivity values that are above the water table and close to the surface, whereas the freshwater

saturated with sand-gravel, and boulder sediments are linked with high resistivity values that are below the water table. The primary aquifer in the region, which is largely on the north and northwestern side of the area, is mainly composed of water-saturated sand-gravel sediments with resistivity values between 20 and 200 Ω.m. The saline sediments are predominantly found on the research area's southern side, with resistivity values < 10 Ω.m above and below the water table. The research region is segmented into saline and fresh water aquifers because of the salinity factor.

To study the fluctuation in resistivity values in the lesser Cholistan, a variety of resistivity maps are prepared at various depths, above and below the water table, and these maps are displayed in Fig. 7a-h at depths of 5-, 25-, 50-, 80-, 100-, 125-, 150-, and 200-m, respectively. These depth levels are chosen after a comprehensive evaluation of the resistivity data to identify the areas with the maximum fluctuation in resistivity. Figure 7a shows the resistivity map at a depth of 5-m, which is above the water table. The resistivity value in the research area ranges from less than 10 Ω.m to more than 200 Ω.m, as shown in Figure 7a. Clayey silt or silty clay sediments are indicated by low resistivity values (<10 Ω.m) at a few locations along the northern, and southern sides. High resistivity readings above the water table near the surface reveal boulders and coarse sand-gravel sediments. Figure 7b displays the confined resistivity map, which spans the ranges of 0 and 200 Ω.m. The northern side of the research region exhibits low-high resistivity values (10-200 Ω.m), as can be seen quite clearly on the map. Resistivity values up to 40 Ω.m suggest silty clay or clayey silt with brackish-slightly good quality ground water, while values < 10 Ω.m below the water table show saline sediments. Sand-gravel with good groundwater is indicated by resistivity values greater than 40 Ω.m. The lesser Cholistan's southern region has values < 20 Ω.m above the water table, which represent clayey silt or silty clay. The resistivity maps for 50, 80, 100, 125, and 150 m depth levels are displayed in Figure 7c–g. As can be observed near VES locations AH-38, YZ-

52, and YZ-58, the clay-silty clay and saline sediments at variable depths indicate the low resistivity value below 20 Ω.m in these maps. The incredibly low resistivity readings in magenta color are taken to indicate the presence of a saline aquifer. These maps represent sites around the VES probes KH-22, AH-40, and HS-22 - 25, which are believed to represent an aquifer of fresh water since they are mostly composed of sand, gravel, and boulder sediments and have high resistivity values between 60 and 200 Ω.m. Maps are interpreted with resistivity values between 20-40 Ω.m and below the water table at different depths to indicate areas with clayey or silty sand and marginally fit quality groundwater. Figure 7-h depicts the resistivity map at a 200-m depth. The lesser Cholistan's northeastern and northwestern regions contain sand and gravel with high resistivity values of 60 to 200 Ω.m. High resistivity readings of 60 to 100 Ω.m have been found in several southern locations close to the YZ-68,69,70,71, and YZ-73, which may yield groundwater of good quality. Resistivity values up to 40 Ω.m suggest saline sediments, clayey silt, silt and silty sand, with brackish and marginally fit ground water. We can see from the resistivity analysis of this research area that the salty water aquifer on the southern side of the region is mostly attributable to the paleochannel dissolved with salt content. The salt content was dissolved and infiltrated to the aquifer during alluvial and fluvial deposition of sediments derived from volcanic rocks and ash exposed in the surrounding area.

Fig. 7a-h*: Maps depicting the distribution of true resistivity across varying depths in two dimensions.*

In order to comprehend the hydrostratigraphy of the research region, the profile A-A′ is chosen on the base map (Fig. 8). Interpolation of resistivity data along the profile AA' yields the resistivity cross-section AA' (Fig. 9a&b). The electrical resistivity processed data and borehole data closest to the profile A-A′ are used to create 2D cross sections (Fig. 9a) along this profile. The profile's interpreted outcomes are displayed in Fig. 10a as a generalized five-layer scenario. Low and high resistivity values appear to fluctuate along the profile AA'. The fresh water is at a very shallow depth, as shown by the high resistivity values between VES sites KH-22 and HS-25. AA's profile's cross-section clearly reveals five layers. Above the water table, which varies roughly from the west to northeast side of the profile, is the first layer, which is composed of dry sediments like silty sand, sand-gravel, and boulders. The second layer exhibits a freshwater aquifer with some high resistivity values on the northeastern side, whereas the western side shows a sand aquifer with brackish quality water between LQ-10 and KH-22. On the western side, between LQ-5 and KH-33, there is a third layer of silty sand, and on the northeastern side, there is a sand aquifer with quality ground water. A fourth layer of clay, silty clay with considerable fine silt, can be observed from the western side, between LQ-10 and KH-22, showing resistivity values of 30 Ω.m. From the western side, a fifth layer of saline sediments of ≤ 10 Ω.m can be depicted sustaining a sand aquifer between LQ-05 and YZ-27 at a depth of 135–170m. While there is sand and gravel with a high resistivity of 100 to 200 Ω.m on the northeastern side between KH-33 - HS-25, which may yield good-quality groundwater. A typical stacked representation of 2D depth maps is generated to clearly depict the distribution of lithologies and aquifer conditions with depths up to 200 Ω.m. (Fig. 10).

Fig. 8: *Base map of the study area showing VES points, boreholes, and cross section AA'.*

Fig. 9: a) *Electrical resistivity distribution versus depth along cross section AA',* b) *Lithological model of the subsurface along cross section AA'.*

Fig. 10: *Visualizing Water Quality Distribution with Enhanced Precision: Multi-layered Maps Stacked by Resistivity Ranges.*

Conclusions

Groundwater salinity can be determined using a VES survey, which can also be used to detect subsurface aquifer layers. In the study area, VES survey was carried out in 230 pre-selected locations. The groundwater aquifer conditions in the lesser Cholistan area of the district of Bahawalpur were evaluated spatially using the VES findings and lithological logs of boreholes. In the research area, three to five geo-electric subsurface layers with varying thicknesses are evaluated as an outcome of processing geophysical data. A number of resistivity maps are generated at different depths, both above and below the water table. The distribution of fresh and saline water aquifers in the study area is determined by comparing all of the electrical resistivity maps. The modeling and re-inversion of 1D electrical resistivity data collected in the lesser Cholistan delineated the boundary between the saline water and freshwater zones, with remarkable achievement. Borehole data from several localities throughout the region were used to map the near surface lithology and to calibrate the modeled resistivity curves. At each resistivity point, the value of the formation resistivity was calculated and validated using the lithologies of drilled boreholes. The created 2D resistivity surface maps enabled in describing the fresh and saline water

zones at various depth levels. Very low resistivity measurements in the southern portion indicated the occurrence of a saline water aquifer with clay rich sediments. On the other hand, the comparatively high resistivity values with sand and gravel deposits in the northern segment of the lesser Cholistan strongly indicated the presence of fresh groundwater.

References

Samson, S., Elangovan, K. (2015). Delineation of groundwater recharge potential zones in Namakkal District, Tamilnadu, India using remote sensing and GIS. *J Indian Soc Remote Sens*, **43**(4),769–778.

Beaumont C, Foreland basins. Geophysics J. R Astr Soc. 1981; 65: 291-329.

Bhutta AT, Cleves MA, Casey PH, Cradock MM, Anand KJ, Cognitive and behavioral outcomes of school-aged children who were born preterm: A meta-analysis. Jama. 2002; 288: 728-37. https://doi.org/10.1001/jama.288.6.728.

Bowling CJ, Harry DL, Rodriguez BA, Zheng C, Integrated geophysical and geological investigation of heterogeneous fluvial aquifer in Columbus Mississippi. Journal of Applied Geophysics, 2007; 62: 58-73.

Bowling CJ, Rodriguez BA, Harry DL, Zheng C, Delineating alluvial aquifer heterogeneity using resistivity and GPR data. Ground Water, 2005; 43: 890-903.

Burbank DW, Rayonlds RGH, Stratigraphic keys to the timing of deformation; An example from the northwestern Himalayan foredeep, (Paola, C. and Kleinspehn, K. ed,) In New perspectives in basin analysis: New York. Springer-Verlag, 1988; 331-351.

Burbank DW, Tahirkheli RAK, The magnetostratigraphy, fission-track dating and stratigraphic evolution of the Peshawar intermontane basin, northern Pakistan. Bulletin of the Geological Society of America, 1985; 96: 539-552.

Burbank DW, The chronology of intermontane-basin development in the northwestern Himalaya and the evolution of northwest syntaxes. Earth and Planetary Science Letters, 1983; 64: 77-92.

Courteaud M, Ritz M, Robineu B, Join JL, Coudray J, New geological and hydrogeological implications of the resistivity distribution inferred from audiomagnetotellurics over La Fournaise young shield volcano (Reunion Island). Journal of Hydrology, 1997; 203: 93–100.

Currell MJ, Drawdown "Triggers": A Misguided Strategy for Protecting Groundwater-Fed Streams and Springs. Groundwater, 2016; 54: 619–622.

Edmund A, Hydrogeological deductions from geoelectric survey in Uvwiamuge and Ekakpamre communities, Delta State, Nigeria. International Journal of Physical Sciences, 2009; 4: 477–485.

Farid A, Jadoon K, Akhter G, Iqbal MA, Hydrostratigraphy and hydrogeology of the western part of Maira area, Khyber Pakhtunkhwa: A case study by using electrical resistivity. Environmental Monitoring and Assessment, 2013; 185: 2407–2422.

Farid A, Khalid P, Jadoon KZ, Jouini MS, The depositional setting of the late quaternary sedimentary fill in southern Bannu basin, northwest Himalayan fold and thrust belt. Pakistan Environmental Monitoring Assessment. 2014; 186: 6587-6604.

Fetter CW, Applied Hydrogeology, Third Edition, Prentice-Hall Publishing Co. New York, 1994; 691.

Geyh MA, Ploethner D, Origin of a fresh groundwater body in Cholistan, Thar Desert, Pakistan. Geological Society, London, Special Publication, 2015; 99-109.

Hasan M, Shang Y, Akhter G, Khan M, Geophysical investigation of fresh saline water interface: A case study from South Punjab, Pakistan. Ground Water. 2017; 55: 841-856. https://doi.org/10.1111/gwat.12527.

Hassan SB, Groundwater Hydrology of Bahawalpur Area, West Pakistan, WAPDA (WASID), 1965; Pub. No.44.

Kamal MT, Shamsi RA, Geohydrology of the Bahawalpur Project area, West Pakistan. WAPDA (WASID), 1965; Pub. No.44.

Khalid P, Ullah S, Farid A, Application of electrical resistivity inversion to delineate salt and freshwater interfaces in quaternary sediments of northwest Himalaya, Pakistan. *Arab J Geosci*. 2018; 11**:** 112. https://doi.org/10.1007/s12517-018-3471-0.

Kosemani SE, Oyelami T, Assessment of heavy metals contamination of groundwater in Ila Orangun community, Osun State, Nigeria. Int. J. Biol. Res. 2017; 2: 32-36.

Mondal NC, Singh V P, Ahmed S, Delineating shallow saline groundwater zones from Southern India using geophysical indicators. Environmental Monitoring and Assessment, 2013; 185: 4869-4886.

Muchingami I, Hlatywayo DJ, Nel JM, Chuma C, Electrical resistivity survey for groundwater investigations and shallow subsurface evaluation of the basaltic-greenstone formation of the urban Bulawayo aquifer. Physics and Chemistry of the Earth, 2012; 50: 44-51.

Muhammad , Ehsan MI Khalid P, Optimizing exploration of quality groundwater through geophysical investigations in district Pakpattan, Punjab, Pakistan. *Arab J Geosci*. 2022; 15: 721. https://doi.org/10.1007/s12517-022-09990-8

Muhammad S, Ehsan MI, Khalid P, Sheikh A, Hydrogeophysical modeling and physio-chemical analysis of quaternary aquifer in central part of Bari Doab, Punjab, Pakistan. *Model. Earth Syst. Environ.* 2022.

NESPAK-Ilaco, Salinity control and reclamation project, Scarp VI, Final plan. Geohydrologtt, 1980; 2: 1-90.

Okoro EI, Egboka BCE, Onwuemesi AG, Evaluation of aquifer characteristics of Nanka Sands using Hydrogeological method in combination with Vertical Electrical Sounding (VES). Journal of Applied Science and Environmental Management, 2010; 14: 5-9.

Pakistan Water Partnership (PWP), Supplement to the framework for actions (FFA) for Achieving the Pakistan Water Vision 2025, 2001.

Qureshi, AS, McCornick PG, Sarwar A, Sharma BR, Challenges and prospects of sustainable groundwater management in the Indus Basin, Pakistan. Water Resour. Manag., 2010; 24: 1551–1569. https://doi.org/10.1007/s11269-009-9513-3.

Qureshi AS, McCornick PG, Qadir M, Aslam Z, Managing salinity and water logging in the Indus Basin of Pakistan. Agric. Water Manag., 2008; 95: 1–10. https://doi.org/10.1016/j.agwat.2007.09.014.

Rasool A, Farooqi A, Masood S, Hussain K, Arsenic in groundwater and its health risk assessment in drinking water of Mailsi, Punjab, Pakistan. Human Ecol. Risk Assess. Int. J., 2016; 22: 187-202. https://doi.org/10.1080/10807039.2015.1056295.

Riddell ES, Lorentz SA, Kotze DC, A geophysical analysis of hydro-geomorphic controls within a headwater wetland in a granitic landscape, through ERI and IP. Hydrology and Earth Systems Sciences, 2010; 14: 1697-1713.

Schrott L, Sass O, Application of field geophysics in geomorphology: advances and limitations exemplified by case studies. Geomorphology, 2008; 93: 55-73.

Shamsi RA, Hamid A, Khan ZA, Quality of groundwater in Bahawalpur Area. WAPDA (WASID), Pub. No.42. 1965.

Singh G, Joshi RD, Singh AB, Stratigraphic and radiocarbon evidence for the age and the development of the three salt lake deposites in Rajasthan, India. Quaternary Research, 1972; 2: 496-505.

Singh G, Joshi RD, Chopra SK, Singh AB, Late Quaternary history of vegetation and climate of the Rajasthan desert, India. Philosophical Transactions of the Royal Society of London, 1974; 267: 567-501.

Stewart M, Layton M, Lizanec T, Application of Resistivity Surveys to Regional Hydrogeologic Reconnaissance. Ground Water, 21; 1983: 42-48.

Sultan SA, Mekhemer HM, Santos FAM, AbdAlla M, Geophysical Measurements for subsurface mapping and groundwater exploration at the central part of the Sinai Peninsula, Egypt. The Arabian Journal for Science and Engineering, 34; 2014: 103–119.

Utom AU, Odoh BI, Okoro AU, Estimation of aquifer transmissivity using Dar Zarrouk Parameters derived from surface resistivity measurements: A case history from parts of Enugu town (Nigeria). Journal of Water Resources and Protection, 2012; 04: 993-1000.

Vouillamoz JM, Hoareau J, Grammare M, Caron D, Nandagiri L, Legchenko A, Quantifying aquifer properties and freshwater resource in a coastal barrier: a hydrogeophysical approach applied to Sasihithlu (Karnataka state, India). Hydrology and Earth System Science, 2012; 16: 4387-4400.

Wasson RJ, Smith GI, Agrarwal DP, Late Quaternary sediments, minerals, and Inferred geochemical history of Didwana Lake, Thar Desert, India. Palaeogeography, Palaeoclimatology, Palaeoecology, 1984; 46: 345-372.

Wilhelmy H, Das Urstromtal am Ostrand der Indusebene und das Sarasvati-Problem. Zeitschrift filir geomorphologie, Neue Folge, Supplement, 1969; 8: 76-93.

Yang YD, Liu MR, Anwar H, Zuo, Yang Y, Impact of future climate change on wheat production in relation to plant-available water capacity in a semi arid environment. Theo. Appl. Climatol., 2014; 115: 391-410.

Yu W, Yang YC, Savitsky A, Alford D, Brown C, Robinson SW, The Indus Basin of Pakistan: The impacts water and agriculture. The World Bank, Washington. 2013, https://doi.org/10.1596/978-0-8213-9874-6.

Zananiri I, Memou T, Lachanas G, Vertical electrical sounding (VES) survey at the central part of Kos island, Aegean, Greece. Geosciences 2006: 411–413.

Zouhri L, Gorini C, Mania J, Deffontaines B, Zeroulai A, Spatial distribution of resistivity in the hydrogeological systems, and identification of the catchment area in the Rharb basin, Morocco. Hydrological Sciences-Journal-des Sciences Hydrologiques, 2004; 49: 387-398.

Click here to access/download;Figure;Fig. 1.jpg

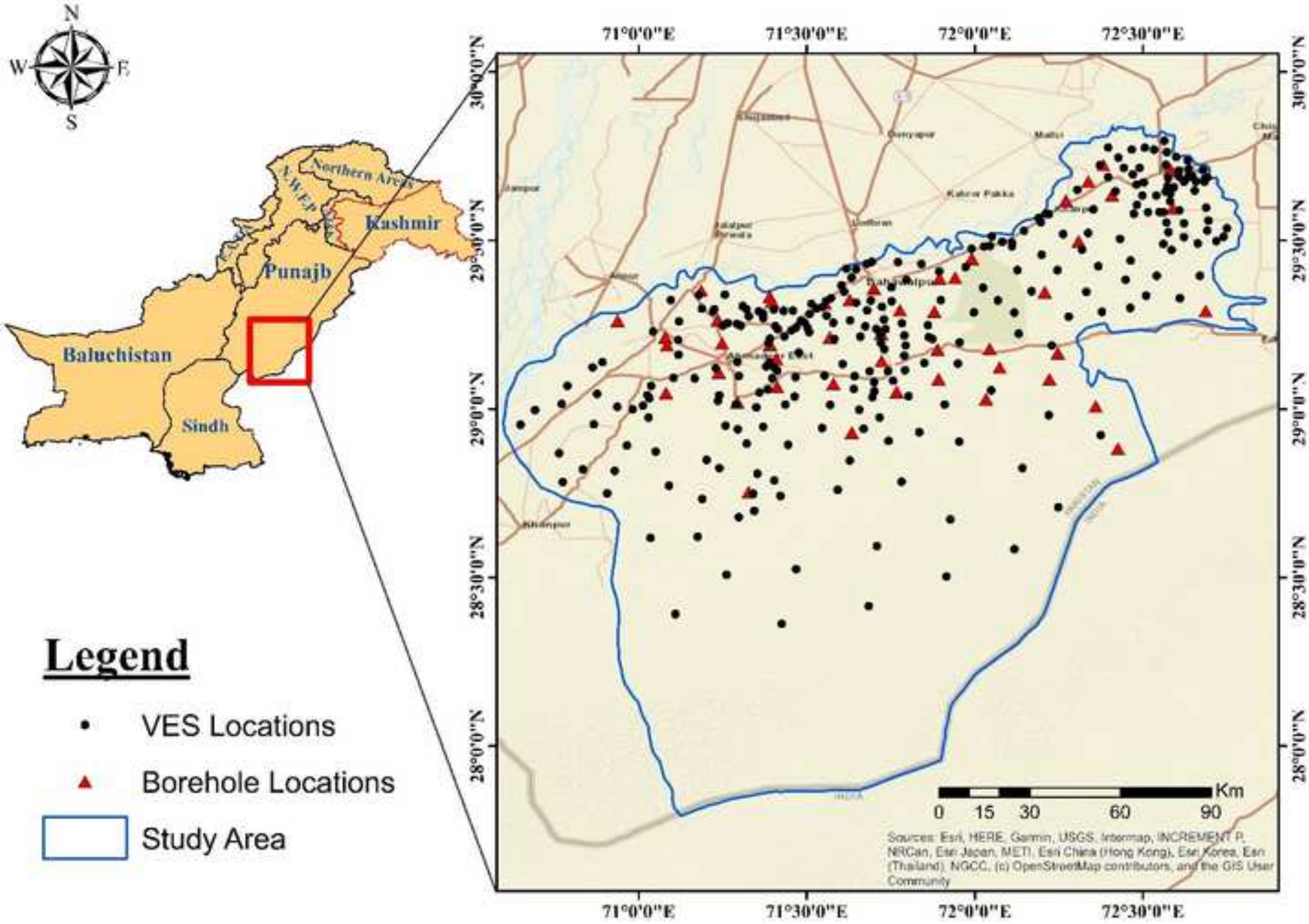

Click here to access/download;Figure;Fig. 2.jpg

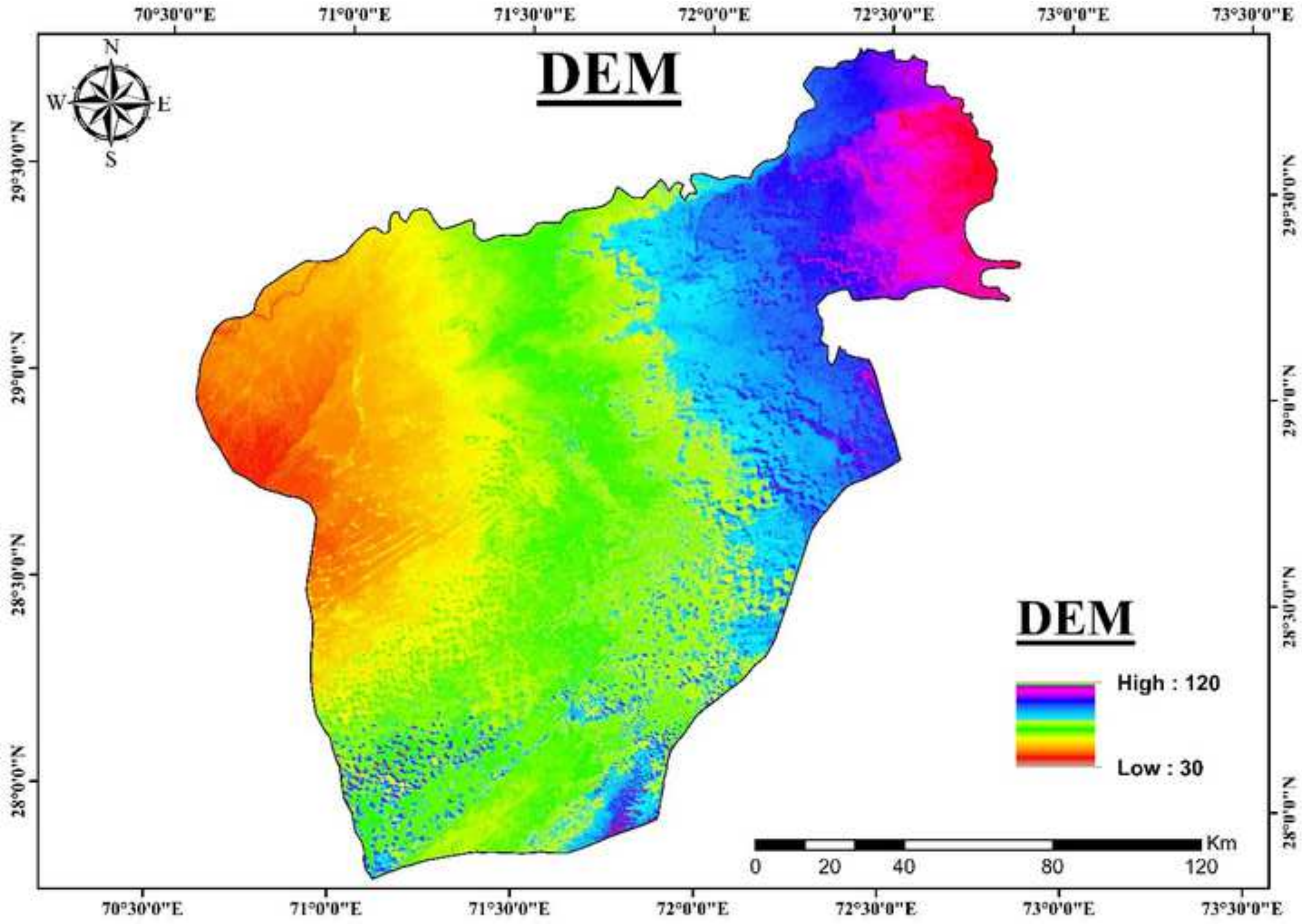

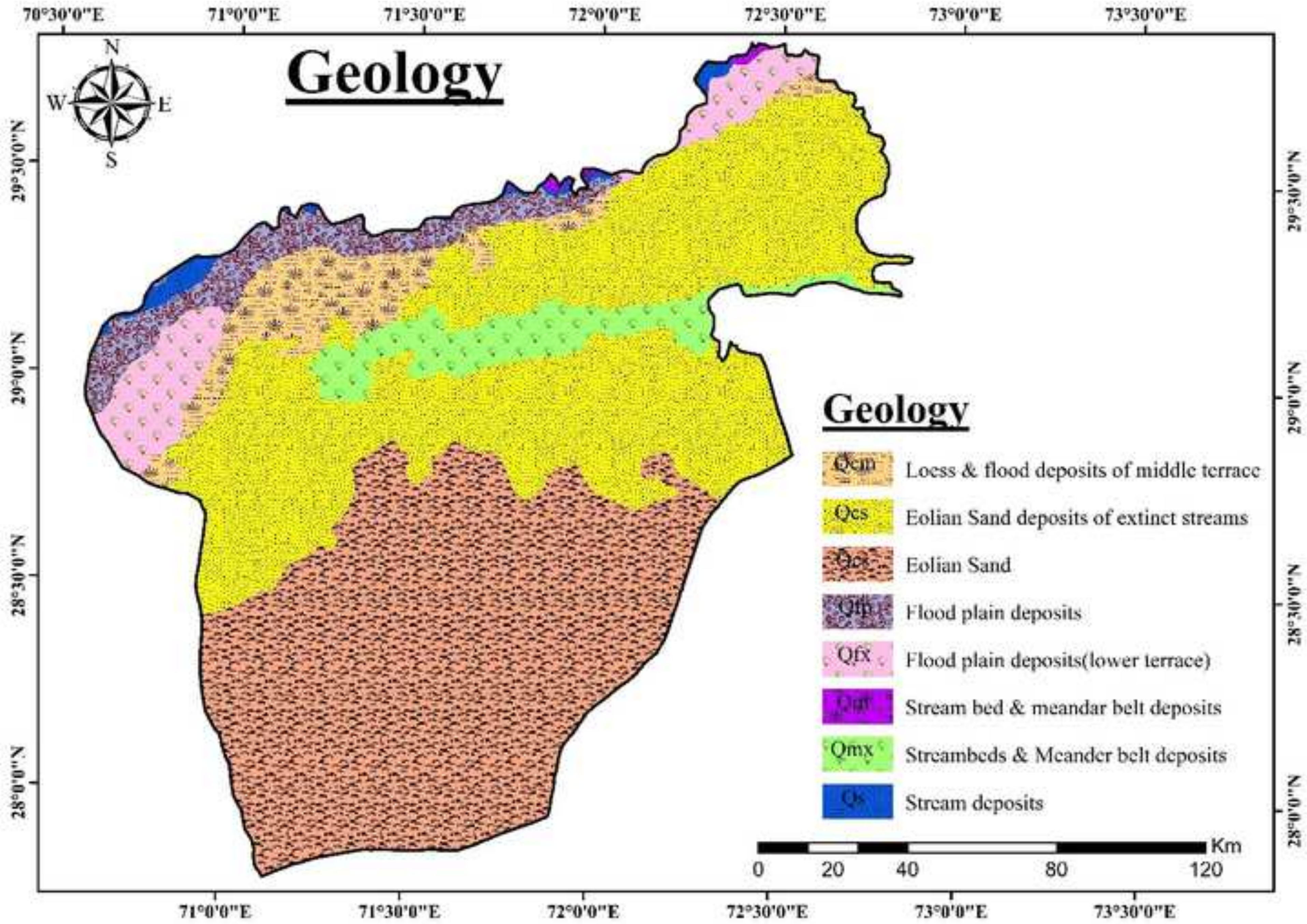

Geology
N
S
W
E
70°30'0"E
71°0'0"E
71°30'0"E
72°0'0"E
72°30'0"E
73°0'0"E
73°30'0"E
29°30'0"N
29°0'0"N
28°30'0"N
28°0'0"N
Geology
Qcm Loess & flood deposits of middle terrace
Qes Eolian Sand deposits of extinct streams
Qes Eolian Sand
Qfp Flood plain deposits
Qfx Flood plain deposits(lower terrace)
Qaf Stream bed & meandar belt deposits
Qmx Streambeds & Meander belt deposits
Qs Stream deposits
Km
0 20 40 80 120

Click here to access/download;Figure;Fig. 4.jpg

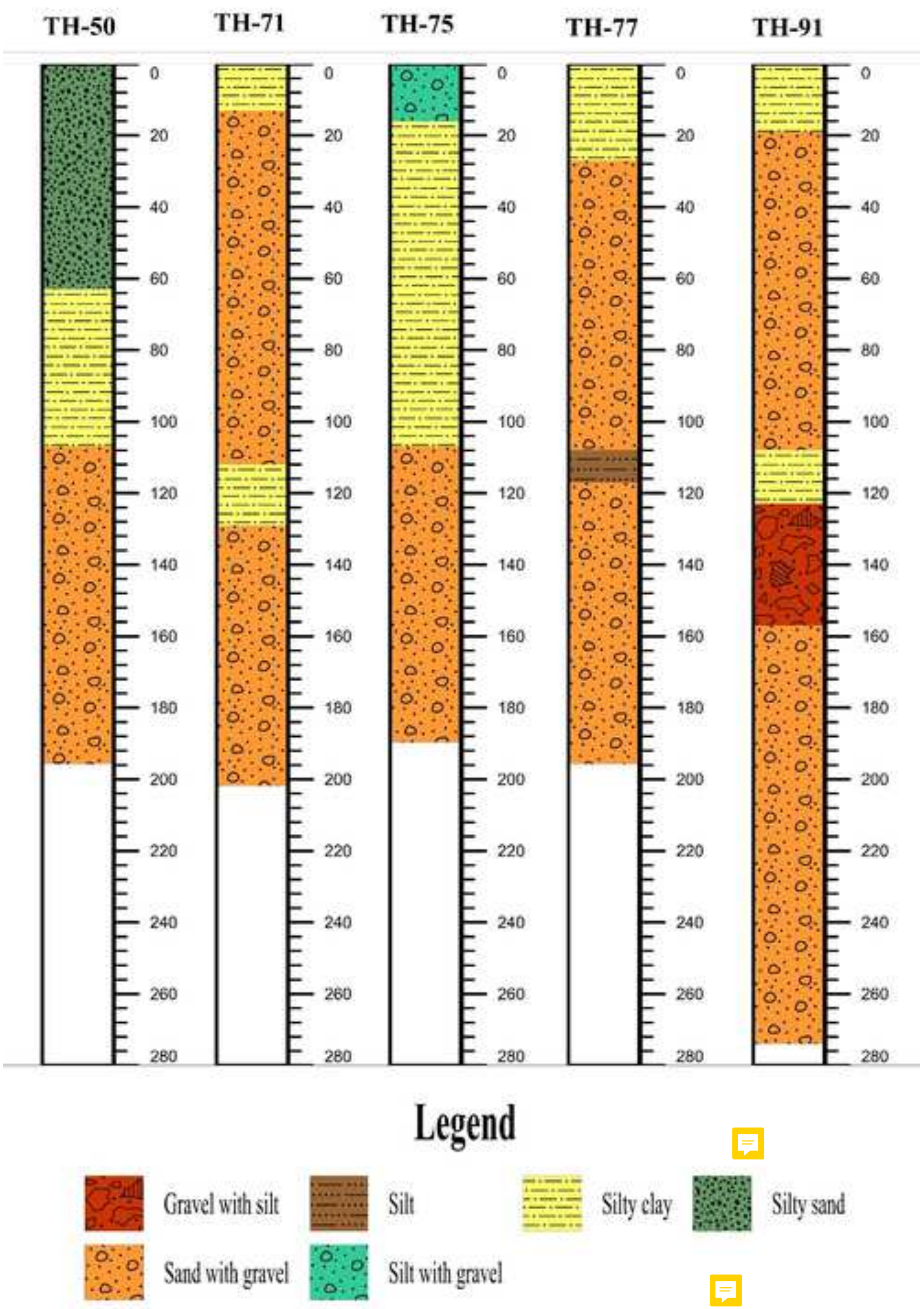

Click here to access/download;Figure;Fig. 5.jpg

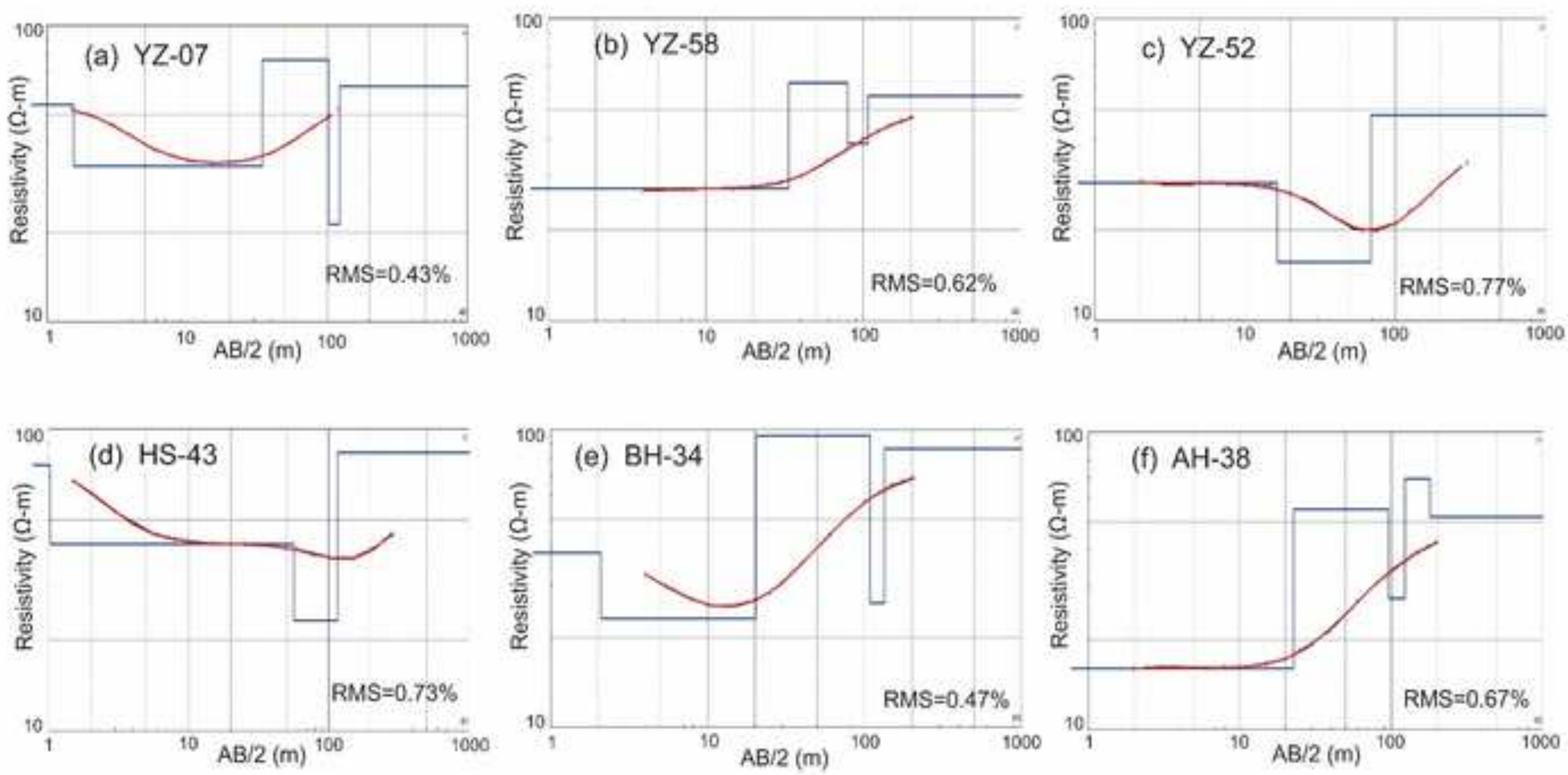

Click here to access/download;Figure;Fig. 6.jpg

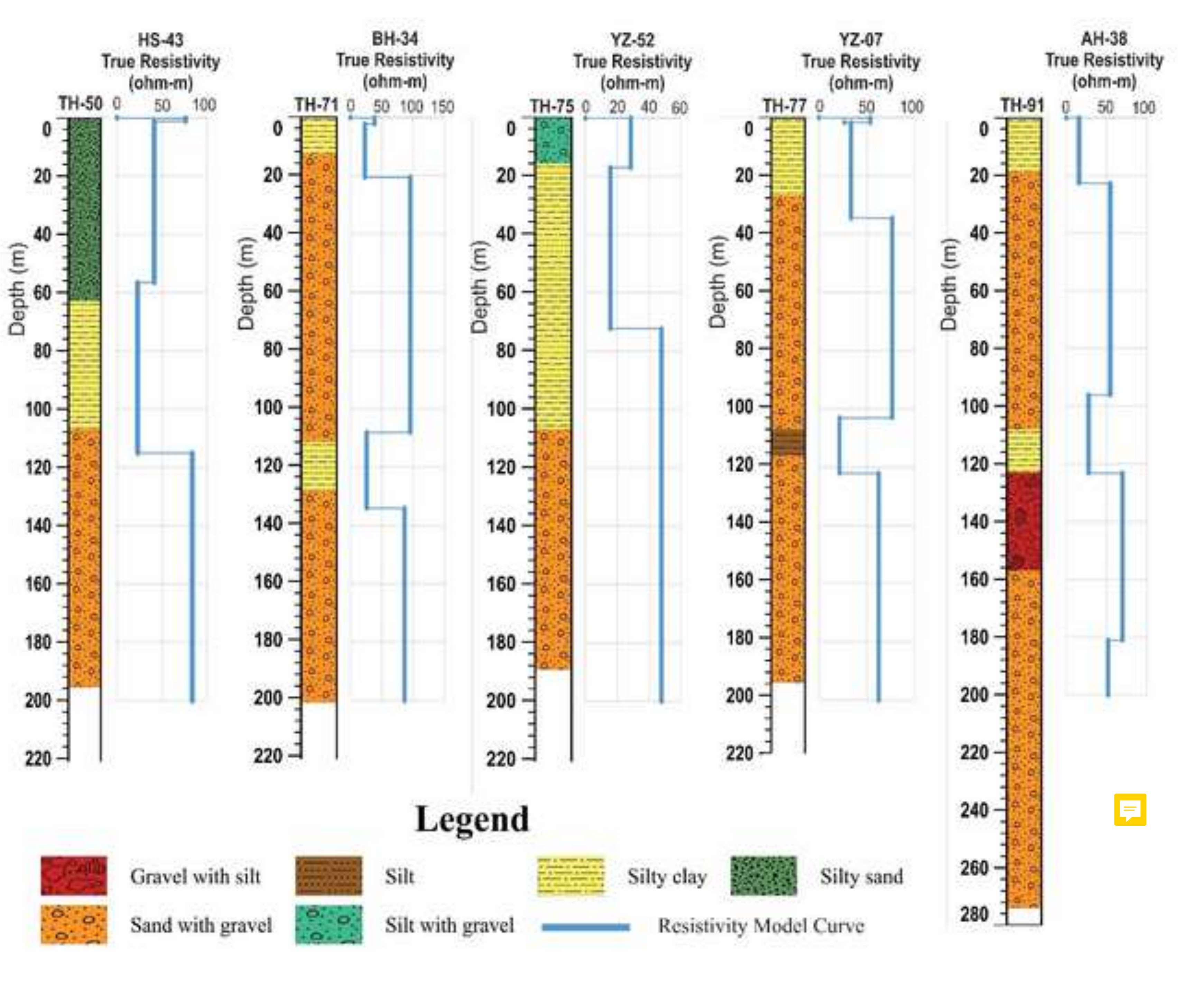

Click here to access/download;Figure;Fig. 7.jpg

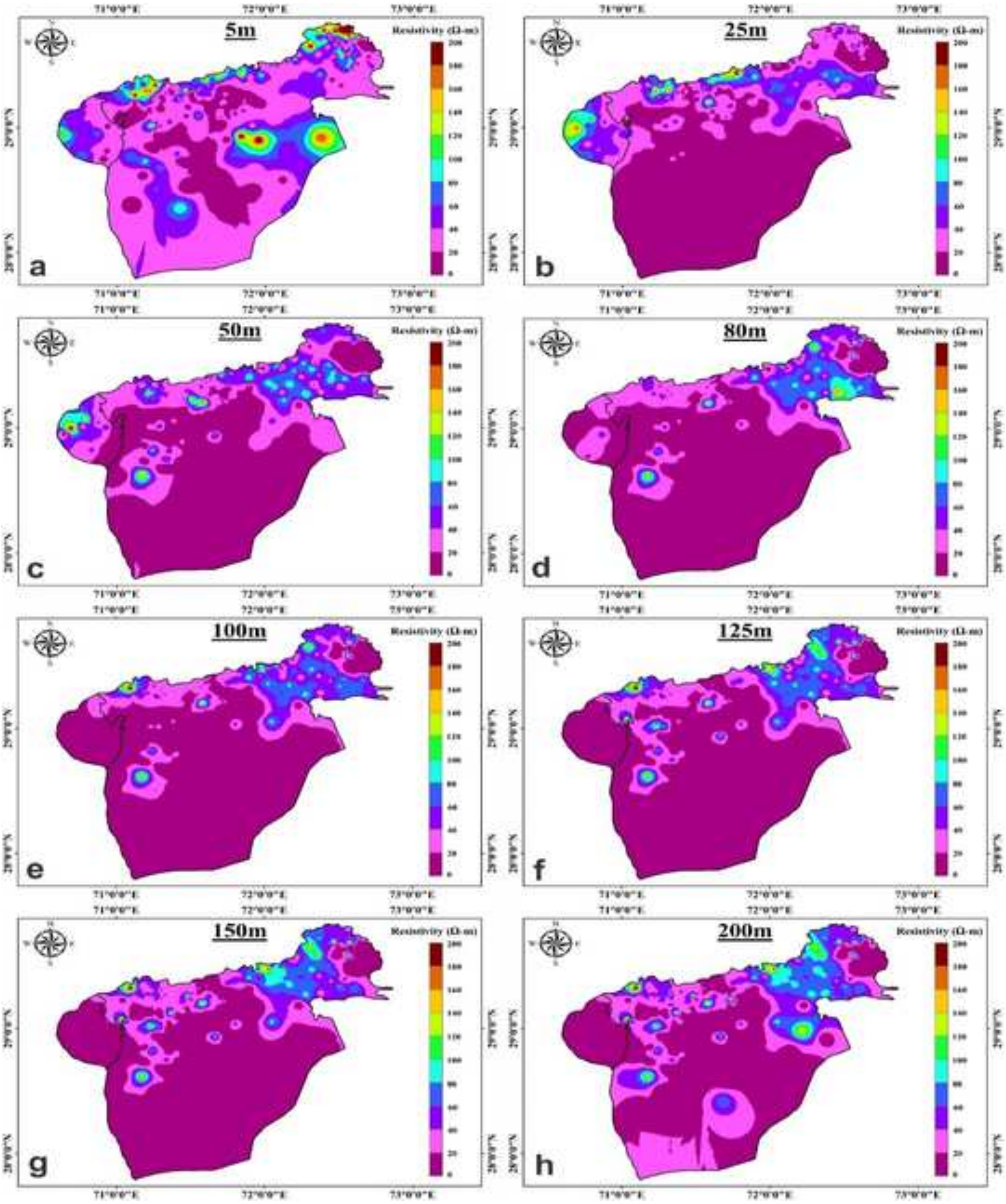

Click here to access/download;Figure;Fig. 8.jpg

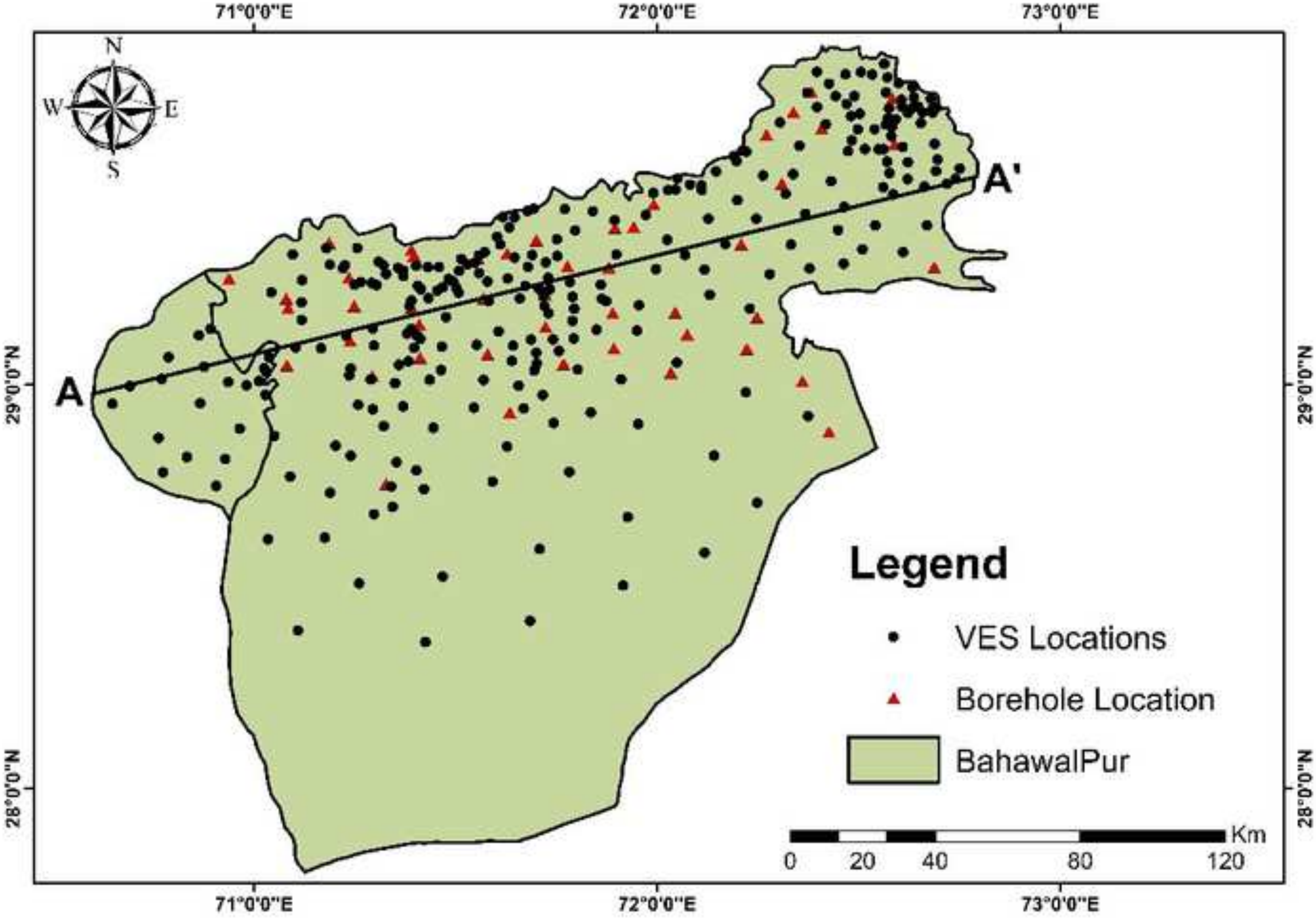

Click here to access/download;Figure;Fig. 9.jpg

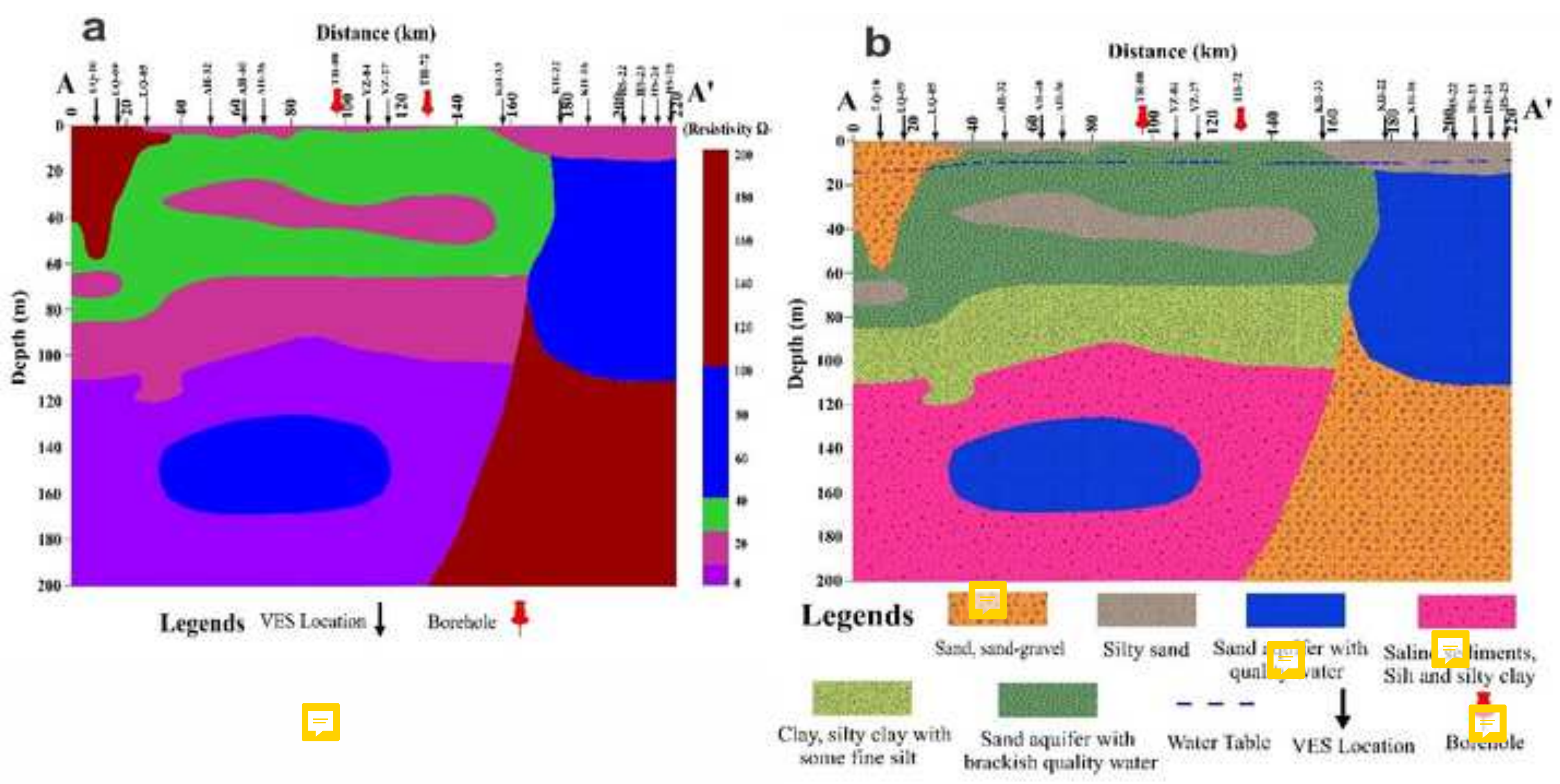

Click here to access/download;Figure;Fig. 10.jpg

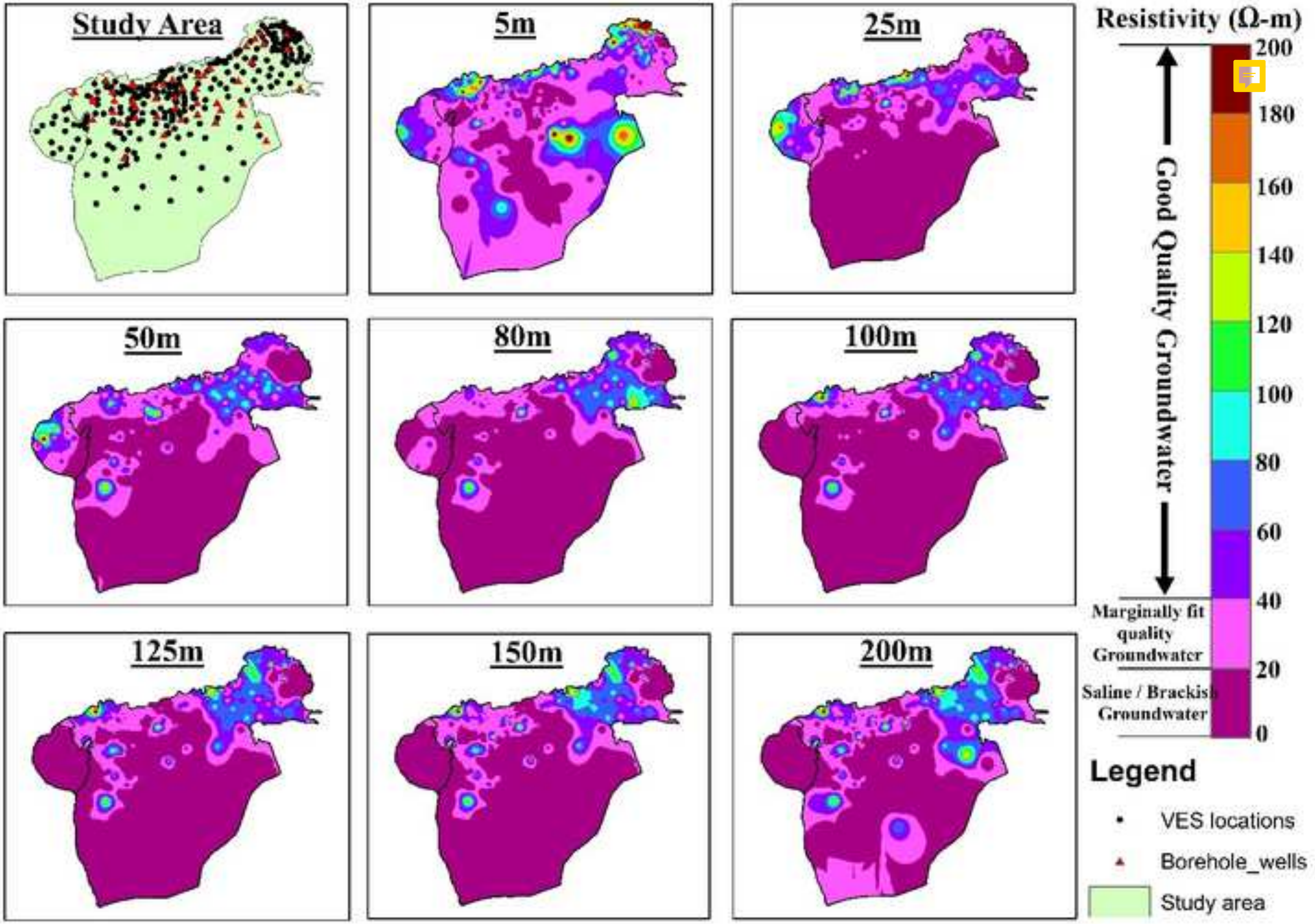

Supplement: Attachment — Submitted filename: PONE-D-23-07514_reviewer.pdf [file pone.0292035.s005.pdf]
