# Supplementary material for: Hydrostratigraphy and hydrogeophysical studies to delineate fresh and saline aquifer boundaries in Lesser Cholistan of Pakistan
Source: PLoS One. 2023 Oct 5;18(10):e0292035. doi: 10.1371/journal.pone.0292035 (PMC10553205; doi:10.1371/journal.pone.0292035)
Supplement: Attachment — Submitted filename: 01_Review Report_Water_2266653.docx [file pone.0292035.s003.docx]

**General Comments**

The authors designed the article to study the “Optimizing water distribution in transboundary rivers based on a synthesis-dynamic-harmonious approach: A case study of Yellow River basin, China”. In this paper, authors considered the changes in the total distributable water volume, of new Yellow River water distribution scheme (“19ZQT” water distribution scheme) assumed water distribution of 37 billion m3 and 30 billion m3, and performed some calculations but there are some discrepancies in the article which needs to be improved before final publication.

**Specific Comments**

The article abstracts need to be revised. The methodology and results of the articles are not mentioned in the article.

The introduction section also needs to be improved because the other scientists work related to water policy is not mentioned very clearly in the introduction. Kindly add more relevant studies to improve the article introduction and relevant literature.

The article methodology section is written very poor. The authors did not add the review of the article of previous studies. The article methodology is based on the theory and no practical work is not involved in it. Kindly add the clear methodology of the article.

Kindly explain your methodology through layout for clear understanding of the readers.

Try to explain the water distribution rules through the layout or diagram.

The article results need to be improved and add more graphical results in it.

The article conclusion needs to be revised properly.
