# Supplementary material for: Hydrostratigraphy and hydrogeophysical studies to delineate fresh and saline aquifer boundaries in Lesser Cholistan of Pakistan
Source: PLoS One. 2023 Oct 5;18(10):e0292035. doi: 10.1371/journal.pone.0292035 (PMC10553205; doi:10.1371/journal.pone.0292035)
Supplement: Attachment — Submitted filename: PONE-D-23-07514_reviewer.docx [file pone.0292035.s004.docx]

<https://www.editorialmanager.com/pone/download.aspx?id=32815734&guid=035cafd6-1852-4c0d-89e4-7fd0aefe6daa&scheme=1>Manuscript	[Click here to access/download;Manuscript;paper 1.docx](https://www.editorialmanager.com/pone/download.aspx?id=32815734&guid=035cafd6-1852-4c0d-89e4-7fd0aefe6daa&scheme=1)

## 1 Hydrostratigraphy and Hydrogeophysical studies to delineate fresh and saline aquifer

2 **boundaries in Lesser Cholistan of Pakistan**
3 Kashif Arif^1^, Perveiz Khalid^1^
4 ^1^Institute of Geology, University of the Punjab 54590 Lahore, Pakistan
5 *Email: [perveiz.geo@pu.edu.pk](mailto:perveiz.geo@pu.edu.pk)

## 6 Abstract

7 The differentiation of saline water and fresh water interfaces is the a key objective in ground water
8 exploration and management. Bahawalpur is the twelfth biggest metropolitan area of Pakistan situated
9 in south Punjab near the bank of River Sutlej and lies at 29°59’55” N latitude and
10 73°15’12” E longitude at an elevation of 521 ft AMSL in the Cholistan area close to the Thar
11 Abandon where a reliable~~continuous~~ supply of fresh water is challenging because of high salinity values in
12 groundwater. Vertical electrical resistivity and borehole data are used to mark fresh and saline
13 interfaces in groundwater. To achieve the results, 230 vertical electric soundings were performed in
14 the study area. A total of 3 to 5 geo-electric layers are identified with modeling along with the
15 processing and interpretation of resistivity data. Bore hole data is used to interpret subsurface
16 lithologies and to calibrate the modeled resistivity curves. The electric resistivity data indicates
17 that thick layers of Quaternary sediments primarily composed
18 of clay, silt, sand, gravels and some kankar are present in the subsurface. Inversion techniques are applied to generate 2D and 3D
19 subsurface resistivity maps to delineate fresh and saline water zones. The generated 2D resistivity
20 maps at variable depths above and below water table and formation resistivity maps are successfully
21 utilized to differentiate fresh and saline water zones. The identification of a saline water aquifer
22 within sediments rich in clay was made possible by the observation of very low resistivity
23 measurements in the southern region. Conversely, the detection of relatively high resistivity
24 values, coupled with the presence of sand and gravel deposits in the northern section of the lesser
25 Cholistan area, provided compelling evidence of the existence of fresh groundwater. These
26 findings have significant implications for the management of water resources in the region, as they
27 provide valuable insights into the distribution and availability of groundwater resources for future
28 use.
29 **Keywords** Electrical resistivity, Saline groundwater, Quaternary sediments, Cholistan

## 30 Introduction

31 The sustainable supply of freshwater is one of the key parameters in urbanization and the
32 socio-economic development of an area (Muhammad et al., 2022). Surface water, which includes
33 streams, canals, lakes, rivers, etc. and groundwater are two primary cheap and easily available
34 sources of freshwater ([Hasan, 2017](http://researcherslinks.com/current-issues/Evaluation-of-Ground-Water-Quality-for-Irrigation/24/1/1149/html#Hasan--M.--Y.-Shang--G.-A)). Due to high solubility of different solutes water is capable to
35 dissolve suspend, soak and adsorb various impurities such as arsenic, sulfides, and chlorides,
36 organic and inorganic etc. ([Kosemani and Oyelami, 2017](http://researcherslinks.com/current-issues/Evaluation-of-Ground-Water-Quality-for-Irrigation/24/1/1149/html#Kosemani--S.E.-an)). Pakistan is among those countries that
37 are facing freshwater scarcity. The whole country is situated in arid to semi-arid regions with
38 low precipitation. The majority of the population relies on groundwater for their domestic,
39 agricultural and industrial needs. The over-extraction of groundwater is one of the main ~~reason in~~
40 ~~enhancing~~ causes of increasing salinity in the groundwater whereas the poor dumping strategies of solid waste is the
41 main source of groundwater contamination ([Rasool, 2017](http://researcherslinks.com/current-issues/Evaluation-of-Ground-Water-Quality-for-Irrigation/24/1/1149/html#Rasool--A.--A.-Farooqi--S.-M)).
42 Cholistan desert – a part of Thar desert – covers an area of 25,800 km^2^ and is the largest desert
43 in the southern Punjab province of Pakistan (Figure 1). This desert is divided into two parts: the Lesser
44 Cholistan and the Higher Cholistan. Around 16,000 km^2^ of the
45 district is comprised of the Cholistan Desert that is covered by dunes of low height (Farooq et al.,
46 2001). The density of dunes increases towards the northwest. Bahawalpur city is the largest city in the Lesser Cholistan while
47 the Higher Cholistan is uninhabited with high sand dunes upto 100 m. The economy of this district is mainly
48 based on agriculture thus the continuous supply of freshwater is the backbone of its economy.
49 However, the existing groundwater is highly contaminated by hazardous pollutants. The analysis
50 of groundwater performed by PCRWR from 2002 to 2015 demonstrates that the groundwater in
51 most of the localities in this district is not safe for drinking. In the southern part of the Bahawalpur
52 area, between Fort Abbas and Derawar Fort, there is a zone of reduced salinity. This relatively
53 low-lying area corresponds to the ancient channel of the Ghaggar (Hakra) River. Although the
54 Ghagger (Hakra) channel is now generally dry, floodwaters occasionally flow through it from the
55 east, near Fort Abbas, in periods of uncommonly high rainfall. Available data indicate that the
56 upper part of the aquifer along this channel contains water of about 4,000 ppm TDS. A deep-water
57 sample (~ 410 m depth) collected near to Derawar Fort contained high salt concentrations up to
58 25,400 ppm (Geyh and Ploethner, 2008).

59

60	Fig. 1: *Base map of the study area showing VES and Borehole locations.*

61

62 The study area is a part of the Punjab Platform that is a subdivision of the Upper Indus
63 Basin. The study area consists of alluvial andaeolian plains (Farooq et al., 2001). The area along the
64 Sutlej River comprises the alluvial plain whereas dunes cover the aeolian plain of the
65 Cholistan Desert. The planning and management for better quality water supplies are
66 inefficient in Bahawalpur City (Anwar and Bureste, 2011). The use of hydrogeological
67 investigations aided by appropriate geophysical techniques to assess the
68 groundwater potential in the confined or unconfined aquifer systems in a particular area are very
69 common (Courteaud et al., 1997; Zouhri et al., 2004; Schrott and Sass, 2008; Edmund, 2009;
70 Okoro et al., 2010; Riddell et al., 2010; Muchingami et al., 2012; Utom et al., 2012; Vouillamoz
71 et al., 2012; Farid et al., 2013; Mondal et al., 2013; Farid et al., 2014). The spatial and vertical
72 distribution of different characteristics of the aquifer such as facies distributions and the variation of hydraulic
73 parameters are fundamental for the study of alluvial deposits.
74 Since the saline water has an enormous amount of total dissolved solids (TDS),
75 modeling and delineation of fresh and saline water boundaries in the subsurface are necessary
76 before planning depths and locations ofwater extraction wells and tube wells. The primary objective of this
77 study is to delineate fresh and saline water interfaces and to map the fresh water aquifer boundaries.

## 78 Geology and Geomorphology of the Area

79 Pakistan is situated at the collisional boundary of the Indian and Eurasian Plates, which creates
80 a significant number of intermontane basins and valley systems (Farid et al., 2018). The Indus basin is
81 one of several? basins produced by the action of tectonic activity. Sediments generated
82 by the erosion of the surrounding mountains are transported into this basin via several(?)
83 sedimentary transport agents. These sediments may vary in size from very coarse alluvial fan
84 gravels and channel deposits with high hydraulic conductivities to very fine lacustrine clays with
85 low hydraulic conductivity. Thus, the grain size of the sediments, lithology
86 and geomorphic nature of the deposited sediments are the important factors in the development of
87 clastic fluvial aquifers and their hydraulic characteristics (Fetter, 1994; Bowling et al., 2005). Since
88 the study area is far away from the source of the sediments mainly fine-grained silt/clay to medium
89 to coarse-grained sand are present.
90 The borehole data suggests that a 457 m thick cover of alluvial sediments comprising
91 fine-grained silt and clay to medium to coarse sand of Late Quaternary age is present throughout
92 the area (Shamsi et al., 1965). This cover is heterogeneous in nature with significant lateral and
93 horizontal variations in lithology (Kemal et al., 1965; Hassan, 1965). Thus, multiple aquifer
94 systems may be present in the study area. According to NESPAK report (1980), the alluvium is
95 saturated up to 1,000 m depth. No continuous layer of clay is reported above the aquifer, thus an
96 unconfined type of aquifer may be present in the area. However, confined or semi-confined
97 aquifer conditions may prevail locally. The landforms are developed by the action of wind and river and
98 consist of two units: flood plain deposits and aeolian deposits. The width of flood plains varies
99 from 10 – 20 km in different localities. According to geomorphological classification, the study
100 area consists of level plains, channel infills, basin, and levees as shown in Fig. 2.

101

102	Fig. 2: *Digital elevation model shows major geomorphology of the area.*

103

104 The sediments deposited in the area are mainly fine to medium grained silt, clay, and sand in the
105 association of Kanker. The sand plains of the Cholistan Desert are comprised of
106 medium-height sandy ridges and inter-dunal hollows of the Hakra River. These deposits are composed
107 of fine to medium silty sand and silt with low permeability. These grains are well rounded,
108 well-sorted, and transported by wind from adjacent arid zones (Fig. 3 & Fig. 4). An old channel of
109 the Hakra River forms a freshwater channel (Geyh and Ploethner, 2015). In the 16^th^ century, this river
110 ceased flowing (Wilhelmy, 1969) and until the mid-20^th^ century the river flowed only during heavy rainfall events.
111 This ephemeral fluvial environment exhibits a flat, hardpan surface covered by irregular patches of dunes up
112 to10 m high. The upper layer of the floodplain comprises fine-grained silt to sandy loam and silty
113 clays of low permeability but good porosity. Thus, rainwater may accumulate in depressions and
114 may stay for a couple of days to several weeks. Since 1960, some portion of this desert has been

converted into cultivated land after the construction of irrigation canals from Sutlej River.

118

119	Fig. 3: *Geological Map of Cholistan Desert.*

120

115 Rainwater and the Sutlej River are the main recharge sources in the study area. The area
116 receives less than 200 mm of rainfall annually.
121 Due to the
122 lack of permeability at the surface, rainwater does not penetrate deeply into the underlying sediments.
123 The recharge rate can be divided into deep and shallow categories. The paleohydrological
124 situation can be used to explain different recharge rates for shallow and deep aquifer layers. No
125 reliable temporal information is available in the western part of the Cholistan area. However,
126 pluvial conditions existed between 13,000 and about 4000 years ago in the eastern part of the
127 Cholistan (Singh et al. 1972, 1974; Wasson et al.; 1984). According to literature, two pluvial
128 periods may have existed from 13,000 to 8,000 years and 7,000 to 4,000 years (Geyh and
129 Ploethner, 2015).
130 A few studies related to hydrogeophysical investigations and groundwater
131 quality are available in the literature. In 1968, the USGS conducted a study on groundwater hydrology
132 of the Punjab Province. According to this study, Most of the area of district Bahawalpur comprises
133 highly saline water with TDS> 5000 ppm except a narrow freshwater corridor along the Sutlej
134 River and an old channel of Hakra River between Fort Abbas to Derawar Fort. Farooq et al. (2001)
135 conducted a study on physio-chemical analysis of shallow water samples collected from 20 – 70
136 m depth. They concluded that the groundwater is highly mineralized in most of the area of Rahim
137 Yaar Khan District with a high concentration of TDS (27,000 ppm) and high sodium absorption
138 ration ~ 54 mg/l).

139

140	Fig. 4*: Boreholes in the study area representing the shallow subsurface geology.*

141

## 142 Methodology

143 Electrical resistivity has long been used as a method to detect subsurface hydrological and
144 geologic features with considerable success (Stewart et al. 1983; Courteaud et al. 1997;
145 Louis et al., 2002; Zouhri et al., 2004; Riddell et al., 2010). Electrical resistivity depends on both the
146 lithologic makeup of the aquifer and upon the fluid contents of the aquifer. The methodology used in this study
147 involves digitizing (?) existing data comprised of vertical
148 electrical soundings (VES) up to 300 m depth at sparsely distributed locations. For convenience,
149 the VES points were generally collected at distance of 4 to 8 km, mostly along the roads, canals
150 and existing tracks as shown in Fig. 1. A terrameter SAS 4000 is used for VES data acquisition.
151 All VES data were acquired by vertical sounding using a Schlumberger array configuration. The
152 Schlumberger array consists of four electric conductors known as electrodes. The two end
153 conductors are current electrodes and the inside two conductors are the potential electrodes. The
154 potential electrodes are installed at the centre of the electrode array with a small separation,
155 typically less than one-fifth of the spacing between the current electrodes (AB ≥ 5MN). The current
156 electrodes are increased to a greater separation during the survey while the potential electrodes
157 remain in the same position until the observed voltage becomes too small to measure.
158 The data from approximately? 120 test holes and20 test wells up to 300 m depth is available from
159 the Water and Power Development Authority (WAPDA) of Pakistan. More than 230 vertical
160 electrical resistivity sounding (VES) data acquired in different places of the study area were
161 used for the characterization of various subsurface lithologies.
162 VES data is interpreted using a
163 combination of curve matching technique and computer iterative modeling to determine the number of
164 geo-electric layers in the study area. The analysis of VES curves requires that
165 the measured curve be matched with several model curves with each model curve representing
166 different subsurface resistivity distributions. The final model is constrained with the help
167 of VES data, lithology logs, local geological data, and borehole data in study area, we map different
168 depositional zones in the Bahawalpur district to mark the alluvial aquifer systems as well as the
169 salinity of the groundwater. For calibration and verification of VES results with borehole data,
170 some of the VES points are acquired near he existing boreholes/tubewells. All the VES data
171 points were modeled (Fig.5) using computer software IP2Win (IPI2WIN-1D computer
172 program. 2000; Zananiri et al., 2006; Sultan et al., 2009; Akhter et al., 2012; Farid et al., 2013;
173 Farid et al., 2014; Muhammad and Khalid, 2017), using information derived from lithology logs,
174 ground water levels, geologic maps and EC maps. 2 D models are generated and plotted against
175 the resistivity values verses depth and consist of several horizontal layers separated according to
176 discrete band of resistivity as shown in Fig. 5a-f. In general calibration between lithology and
177 resistivity is established using available borehole information and resistivity data and is shown in
178 Table 1 and in Fig. 6.

178

179	Table 1: *Cutoff values of resistivity for different lithologies used in interpretation of VES data.*

| Resistivity  Zone | Resistivity Range  (Ω-m) | Interpreted Lithology |
| --- | --- | --- |
| Very high | Greater than 230 | Coarse sand with gravels and kanker |
| High | Less than 230 to 100 | sand with gravels with some interbeded thin layer  of silt and clay |
| Medium | Less than 100 to 40 | Medium to coarse sand |
| Low | Less than 40 to 20 | Mixture of sand with some silt and clay |

Silty clay / clayey silt (Above GWT)

Very low	Less than 20	Saline sediments (Below GWT)

180

181 The details provided in Table. 1 has been used to analyze all of the VES curves and
182 developing layer models. Each model response curve represents the virtual VES response of
183 horizontally stratified earth layer using a limited number of sedimentary layers (3-5). The base
184 layer in each model extends to undefined depth value.

185

186 Fig. 5a-f: *Apparent resistivity data are marked by small circles. Solid black curve represents the*
187 *apparent resistivity curve. Red curve is the best fitted curve to the apparent resistivity data. Solid*
188 *blue block line is the modeled resistivity (synthetic resistivity). Horizontal axis is the current*
189 *electrode spacing (AB/2) in meters and vertical axis is the resistivity in ohm meters*.

190

191 Fig .6: *Borehole logs and modeled resistivity curves representing the calibration between*
192 *lithology and resistivity data.*

193

## 194 Result and discussion

195 Interpretations are made for three to five geo-electric subsurface layers in the study
196 area. The resistivity and depth/thickness of each layer is distinctive. Electrical resistivity values of
197 each geo-electric layer can be implemented to characterize the probable underlying
198 lithology, including particle size, porosity, and fluid type. A correlation between the
199 interpreted underlying lithology and true resistivities is created by simultaneously examining the
200 processed data from 230 VES probes and lithological logs of boreholes (Table 1). Furthermore,
201 the lithological logs of boreholes closest to the respective VES probes are used to calibrate the true
202 resistivities (Fig. 6).
203 Clay, silt, and sand comprise the alluvial deposits with occasional? gravel and pebbles layers, are encountered in boreholes in the aquifers. The southern portion of the lesser Cholistan has
204 clay-rich sediments as a result of the intrusion of salt contents, and the findings of the resistivity
205 inversion provide confirmation of this. The interpretation of VES results in this area was
206 challenging due to the relatively low difference in resistivity readings between shallow and deep
207 alluvium. Sand and gravel sediments are more prominent in the north and northwestern part of the
208 study area, where they serve as a good aquifer for fresh groundwater, and clay rich
209 sediments are more dominant in the south and southeast, and contain more saline water
210 according to borehole data shown in Fig. 4. The digital elevation map of Cholistan,
211 indicates that the elevation declines from northeast to. The digital elevation map and the water table elevations are interrelated. The ground water
212 level changes across the Lesser Cholistan. In the northeast at well TH-50, the water table is around
213 ≤ 12m deep, while in the southwest borehole TH-91, it is about ≤ 17m deep. As can be seen in Fig.
214 8a-h, the electrical resistivity survey findings indicate that the south portion of the Lesser
215 Cholistan has significantly lower resistivity values than the north side. In the south region of the
216 study area, where the depth of penetration in the VES findings is very low, resistivity data results
217 confirm that higher proportions of clay-rich or salty sediments prevent the electric current from
218 flowing at greater depths. The dry sand, gravel, and boulder sediments are linked with high
219 resistivity values that are above the water table and close to the surface, whereas the freshwater
220 saturated sand-gravel, and boulder sediments are linked with high resistivity values that are
221 below the water table. The primary aquifer in the region, which is largely on the north and
222 northwestern side of the area, is mainly composed of water-saturated sand-gravel sediments with
223 resistivity values between 20 and 200 Ω.m. The saline sediments are predominantly found on the
224 research area's southern side, with resistivity values < 10 Ω.m both above and below the water table.
225 The research region is segmented into saline and fresh water aquifers because of the salinity factor.
226 To study the fluctuation in resistivity values in the lesser Cholistan, a variety of resistivity maps
227 are prepared at various depths, above and below the water table, and these maps are displayed in
228 Fig. 7a-h at depths of 5-, 25-, 50-, 80-, 100-, 125-, 150-, and 200-m, respectively. These depth
229 levels are chosen after a comprehensive evaluation of the resistivity data to identify the areas with
230 the maximum fluctuation in resistivity. Figure 7a shows the resistivity map at a depth of 5-m,
231 which is above the water table. Therefore, the resistivity values reflect mainly lithologic differences. The resistivity value in the research area ranges from less than 10
232 Ω.m to more than 200 Ω.m, as shown in Figure 7a. Clayey silt or silty clay sediments are indicated
233 by low resistivity values (<10 Ω.m) at a few locations along the northern, and southern sides. High
234 resistivity readings above the water table near the surface reveal boulders and coarse sand-gravel
235 sediments. Figure 7b displays the resistivity map of the confined aquifer, which ranges from 0 to 200
236 Ω.m. The northern side of the research region exhibits resistivity values of 10-200 Ω.m (Fig. 7b),
237 . Resistivity values up to 40 Ω.m suggest silty clay or clayey
238 silt with brackish-slightly good quality ground water, while values < 10 Ω.m below the water table
239 show saline sediments. Sand-gravel with good groundwater is indicated by resistivity values
240 greater than 40 Ω.m. The lesser Cholistan's southern region has values < 20 Ω.m above the water
241 table, which represent clayey silt or silty clay. The resistivity maps for 50, 80, 100, 125, and 150
242 m depth levels are displayed in Figure 7c–g. As can be observed near VES locations AH-38, YZ-
243 52, and YZ-58, the clay-silty clay and saline sediments at variable depths indicate
244 resistivity values below 20 Ω.m in these maps. The resistivity readings < 20 ohm-m are assumed to indicate the presence of a saline aquifer. These maps represent sites around the
245 VES probes KH-22, AH-40, and HS-22 - 25, which are believed to represent an aquifer of fresh
246 water since they are mostly composed of sand, gravel, and boulder sediments and have
247 high resistivity values between 60 and 200 Ω.m. Maps are interpreted with resistivity values
248 between 20-40 Ω.m and below the water table at different depths to indicate areas with clayey or
249 silty sand and marginally fit quality groundwater. Figure 7-h depicts the resistivity map at a 200-
250 m depth. The lesser Cholistan's northeastern and northwestern regions contain sand and gravel
251 with high resistivity values of 60 to 200 Ω.m. High resistivity readings of 60 to 100 Ω.m have been
252 found in several southern locations close to the YZ-68,69,70,71, and YZ-73, which may
253 yield groundwater of good quality. Resistivity values up to 40 Ω.m suggest saline sediments,
254 clayey silt, silt and silty sand, with brackish and marginally fit ground water. We can see from the
255 resistivity analysis of this research area that the salty water aquifer on the southern side of the
256 region is mostly attributable to the paleochannel dissolved with salt content. The salt content was
257 dissolved and infiltrated to the aquifer during alluvial and fluvial deposition of sediments derived
258 from volcanic rocks and ash exposed in the surrounding area.

262

263 Fig. 7a-h*: Maps depicting the distribution of true resistivity across varying depths in two*
264 *dimensions.*

265

266 In order to comprehend the hydrostratigraphy of the research region, the profile A-A′ is chosen on
267 the base map (Fig. 8). Interpolation of resistivity data along the profile AA' yields the resistivity
268 cross-section AA' (Fig. 9a&b). The electrical resistivity processed data and borehole data closest
269 to the profile A-A′ are used to create 2D cross sections (Fig. 9a) along this profile. The profile's
270 interpreted outcomes are displayed in Fig. 10a as a generalized five-layer scenario. Low and high
271 resistivity values appear to fluctuate along the profile AA'. The fresh water is at a very shallow
272 depth, as shown by the high resistivity values between VES sites KH-22 and HS-25. AA's profile's
273 cross-section clearly reveals five layers. Above the water table, which varies roughly from the west
274 to northeast side of the profile, is the first layer, which is composed of dry sediments like silty
275 sand, sand-gravel, and boulders. The second layer exhibits a freshwater aquifer with some high
276 resistivity values on the northeastern side, whereas the western side shows a sand aquifer with
277 brackish quality water between LQ-10 and KH-22. On the western side, between LQ-5 and KH-
278 33, there is a third layer of silty sand, and on the northeastern side, there is a sand aquifer with
279 quality ground water. A fourth layer of clay, silty clay with considerable fine silt, can be observed
280 from the western side, between LQ-10 and KH-22, showing resistivity values of 30 Ω.m. From the
281 western side, a fifth layer of saline sediments of ≤ 10 Ω.m can be depicted sustaining a sand aquifer
282 between LQ-05 and YZ-27 at a depth of 135–170m. While there is sand and gravel with a high
283 resistivity of 100 to 200 Ω.m on the northeastern side between KH-33 - HS-25, which may yield
284 good-quality groundwater. A typical stacked representation of 2D depth maps is generated to
285 clearly depict the distribution of lithologies and aquifer conditions with depths up to 200 Ω.m.
286 (Fig. 10).

287

288	Fig. 8: *Base map of the study area showing VES points, boreholes, and cross section AA’.*

289

290 Fig. 9: a) *Electrical resistivity distribution versus depth along cross section AA’,* b) *Lithological*
291 *model of the subsurface along cross section AA’.*

292

293 Fig. 10: *Visualizing Water Quality Distribution with Enhanced Precision: Multi-layered Maps*
294 *Stacked by Resistivity Ranges.*

295

296 **Conclusions**
297 Groundwater salinity can be determined using a VES survey, which can also be used to detect
298 subsurface aquifer layers. In the study area, the VES survey was carried out in 230 pre-selected
299 locations. The groundwater aquifer conditions in the lesser Cholistan area of the district of
300 Bahawalpur were evaluated spatially using the VES findings and lithological logs of boreholes. In
301 the research area, three to five geo-electric subsurface layers with varying thicknesses are
302 evaluated as an outcome of processing geophysical data. A number of resistivity maps are
303 generated at different depths, both above and below the water table. The distribution of fresh and
304 saline water aquifers in the study area is determined by comparing all of the electrical resistivity
305 maps. The modeling and re-inversion of 1D electrical resistivity data collected in the lesser
306 Cholistan delineated the boundary between the saline water and freshwater zones, with remarkable
307 achievement. Borehole data from several localities throughout the region were used to map the
308 near surface lithology and to calibrate the modeled resistivity curves. At each resistivity point, the
309 value of the formation resistivity was calculated and validated using the lithologies of drilled
310 boreholes. The created 2D resistivity surface maps enabled in describing the fresh and saline water
311 zones at various depth levels. Very low resistivity measurements in the southern portion indicated
312 the occurrence of a saline water aquifer with clay rich sediments. On the other hand, the
313 comparatively high resistivity values with sand and gravel deposits in the northern segment of the
314 lesser Cholistan strongly indicated the presence of fresh groundwater.

## 315 References

316 Samson, S., Elangovan, K. (2015). Delineation of groundwater recharge potential zones in
317 Namakkal District, Tamilnadu, India using remote sensing and GIS. *J Indian Soc Remote*
318 *Sens*, **43**(4),769–778.
319 Beaumont C, Foreland basins. Geophysics J. R Astr Soc. 1981; 65: 291-329.
320 Bhutta AT, Cleves MA, Casey PH, Cradock MM, Anand KJ, Cognitive and behavioral outcomes
321 of school-aged children who were born preterm: A meta-analysis. Jama. 2002; 288: 728-
322 37. <https://doi.org/10.1001/jama.288.6.728>.
323 Bowling CJ, Harry DL, Rodriguez BA, Zheng C, Integrated geophysical and geological
324 investigation of heterogeneous fluvial aquifer in Columbus Mississippi. Journal of Applied
325 Geophysics, 2007; 62: 58-73.
326 Bowling CJ, Rodriguez BA, Harry DL, Zheng C, Delineating alluvial aquifer heterogeneity using
327 resistivity and GPR data. Ground Water, 2005; 43: 890-903.
328 Burbank DW, Rayonlds RGH, Stratigraphic keys to the timing of deformation; An example from
329 the northwestern Himalayan foredeep, (Paola, C. and Kleinspehn, K. ed,) In New
330 perspectives in basin analysis: New York. Springer-Verlag, 1988; 331-351.
331 Burbank DW, Tahirkheli RAK, The magnetostratigraphy, fission-track dating and stratigraphic
332 evolution of the Peshawar intermontane basin, northern Pakistan. Bulletin of the
333 Geological Society of America, 1985; 96: 539-552.
334 Burbank DW, The chronology of intermontane-basin development in the northwestern Himalaya
335 and the evolution of northwest syntaxes. Earth and Planetary Science Letters, 1983; 64:
336 77-92.
337 Courteaud M, Ritz M, Robineu B, Join JL, Coudray J, New geological and hydrogeological
338 implications of the resistivity distribution inferred from audiomagnetotellurics over La
339 Fournaise young shield volcano (Reunion Island). Journal of Hydrology, 1997; 203: 93–
340 100.
341 Currell MJ, Drawdown “Triggers”: A Misguided Strategy for Protecting Groundwater-Fed
342 Streams and Springs. Groundwater, 2016; 54: 619–622.
343 Edmund A, Hydrogeological deductions from geoelectric survey in Uvwiamuge and Ekakpamre
344 communities, Delta State, Nigeria. International Journal of Physical Sciences, 2009; 4:
345 477–485.
346 Farid A, Jadoon K, Akhter G, Iqbal MA, Hydrostratigraphy and hydrogeology of the western part
347 of Maira area, Khyber Pakhtunkhwa: A case study by using electrical resistivity.
348 Environmental Monitoring and Assessment, 2013; 185: 2407–2422.
349 Farid A, Khalid P, Jadoon KZ, Jouini MS, The depositional setting of the late quaternary
350 sedimentary fill in southern Bannu basin, northwest Himalayan fold and thrust belt.
351 Pakistan Environmental Monitoring Assessment. 2014; 186: 6587-6604.
352 Fetter CW, Applied Hydrogeology, Third Edition, Prentice-Hall Publishing Co. New York, 1994;

353	691.

354 Geyh MA, Ploethner D, Origin of a fresh groundwater body in Cholistan, Thar Desert, Pakistan.
355 Geological Society, London, Special Publication, 2015; 99-109.
356 Hasan M, Shang Y, Akhter G, Khan M, Geophysical investigation of fresh saline water interface:
357 A	case	study	from	South	Punjab,	Pakistan. Ground	Water.	2017;	55:	841-
358 856. [https://doi.org/10.1111/gwat.12527.](https://doi.org/10.1111/gwat.12527)
359 Hassan SB, Groundwater Hydrology of Bahawalpur Area, West Pakistan, WAPDA
360 (WASID), 1965; Pub. No.44.
361 Kamal MT, Shamsi RA, Geohydrology of the Bahawalpur Project area, West Pakistan. WAPDA

362	(WASID), 1965; Pub. No.44.

363 Khalid P, Ullah S, Farid A, Application of electrical resistivity inversion to delineate salt and
364 freshwater interfaces in quaternary sediments of northwest Himalaya, Pakistan. *Arab J*
365 *Geosci*. 2018; 11**:** 112. https://doi.org/10.1007/s12517-018-3471-0.
366 Kosemani SE, Oyelami T, Assessment of heavy metals contamination of groundwater in Ila
367 Orangun community, Osun State, Nigeria. Int. J. Biol. Res. 2017; 2: 32-36.
368 Mondal NC, Singh V P, Ahmed S, Delineating shallow saline groundwater zones from Southern
369 India using geophysical indicators. Environmental Monitoring and Assessment, 2013; 185:
370 4869-4886.
371 Muchingami I, Hlatywayo DJ, Nel JM, Chuma C, Electrical resistivity survey for groundwater
372 investigations and shallow subsurface evaluation of the basaltic-greenstone formation of
373 the urban Bulawayo aquifer. Physics and Chemistry of the Earth, 2012; 50: 44-51.
374 Muhammad , Ehsan MI Khalid P, Optimizing exploration of quality groundwater through
375 geophysical investigations in district Pakpattan, Punjab, Pakistan. *Arab J Geosci*. 2022; 15:
376 721. <https://doi.org/10.1007/s12517-022-09990-8>
377 Muhammad S, Ehsan MI, Khalid P, Sheikh A, Hydrogeophysical modeling and physio-chemical
378 analysis of quaternary aquifer in central part of Bari Doab, Punjab, Pakistan. *Model. Earth*
379 *Syst. Environ.* 2022.
380 NESPAK-Ilaco, Salinity control and reclamation project, Scarp VI, Final plan. Geohydrologtt,

381	1980; 2: 1-90.

382 Okoro EI, Egboka BCE, Onwuemesi AG, Evaluation of aquifer characteristics of Nanka Sands
383 using Hydrogeological method in combination with Vertical Electrical Sounding (VES).
384 Journal of Applied Science and Environmental Management, 2010; 14: 5-9.
385 Pakistan Water Partnership (PWP), Supplement to the framework for actions (FFA) for Achieving
386 the Pakistan Water Vision 2025, 2001.
387 Qureshi, AS, McCornick PG, Sarwar A, Sharma BR, Challenges and prospects of sustainable
388 groundwater management in the Indus Basin, Pakistan. Water Resour. Manag., 2010; 24:
389 1551–1569. <https://doi.org/10.1007/s11269-009-9513-3>.
390 Qureshi AS, McCornick PG, Qadir M, Aslam Z, Managing salinity and water logging in the Indus
391 Basin	of	Pakistan.	Agric.	Water	Manag.,	2008;	95:	1–
392 10. <https://doi.org/10.1016/j.agwat.2007.09.014>.
393 Rasool A, Farooqi A, Masood S, Hussain K, Arsenic in groundwater and its health risk assessment
394 in drinking water of Mailsi, Punjab, Pakistan. Human Ecol. Risk Assess. Int. J., 2016; 22:
395 187-202. <https://doi.org/10.1080/10807039.2015.1056295>.
396 Riddell ES, Lorentz SA, Kotze DC, A geophysical analysis of hydro-geomorphic controls within
397 a headwater wetland in a granitic landscape, through ERI and IP. Hydrology and Earth
398 Systems Sciences, 2010; 14: 1697-1713.
399 Schrott L, Sass O, Application of field geophysics in geomorphology: advances and limitations
400 exemplified by case studies. Geomorphology, 2008; 93: 55-73.
401 Shamsi RA, Hamid A, Khan ZA, Quality of groundwater in Bahawalpur Area. WAPDA

402	(WASID), Pub. No.42. 1965.

403 Singh G, Joshi RD, Singh AB, Stratigraphic and radiocarbon evidence for the age and the
404 development of the three salt lake deposites in Rajasthan, India. Quaternary Research,
405 1972; 2: 496-505.
406 Singh G, Joshi RD, Chopra SK, Singh AB, Late Quaternary history of vegetation and climate of
407 the Rajasthan desert, India. Philosophical Transactions of the Royal Society of London,
408 1974; 267: 567-501.
409 Stewart M, Layton M, Lizanec T, Application of Resistivity Surveys to Regional Hydrogeologic
410 Reconnaissance. Ground Water, 21; 1983: 42-48.
411 Sultan SA, Mekhemer HM, Santos FAM, AbdAlla M, Geophysical Measurements for subsurface
412 mapping and groundwater exploration at the central part of the Sinai Peninsula, Egypt. The
413 Arabian Journal for Science and Engineering, 34; 2014: 103–119.
414 Utom AU, Odoh BI, Okoro AU, Estimation of aquifer transmissivity using Dar Zarrouk
415 Parameters derived from surface resistivity measurements: A case history from parts of
416 Enugu town (Nigeria). Journal of Water Resources and Protection, 2012; 04: 993-1000.
417 Vouillamoz JM, Hoareau J, Grammare M, Caron D, Nandagiri L, Legchenko A, Quantifying
418 aquifer properties and freshwater resource in a coastal barrier: a hydrogeophysical
419 approach applied to Sasihithlu (Karnataka state, India). Hydrology and Earth System
420 Science, 2012; 16: 4387-4400.
421 Wasson RJ, Smith GI, Agrarwal DP, Late Quaternary sediments, minerals, and Inferred
422 geochemical	history	of	Didwana	Lake,	Thar	Desert,	India.	Palaeogeography,
423 Palaeoclimatology, Palaeoecology, 1984; 46: 345-372.
424 Wilhelmy H, Das Urstromtal am Ostrand der Indusebene und das Sarasvati-Problem. Zeitschrift
425 filir geomorphologie, Neue Folge, Supplement, 1969; 8: 76-93.
426 Yang YD, Liu MR, Anwar H, Zuo, Yang Y, Impact of future climate change on wheat production
427 in relation to plant-available water capacity in a semi arid environment. Theo. Appl.
428 Climatol., 2014; 115: 391-410.
429 Yu W, Yang YC, Savitsky A, Alford D, Brown C, Robinson SW, The Indus Basin of Pakistan:
430 The	impacts	water	and	agriculture.	The	World	Bank,	Washington. 2013,
431 [https://doi.org/10.1596/978-0-8213-9874-6.](https://doi.org/10.1596/978-0-8213-9874-6)
432 Zananiri I, Memou T, Lachanas G, Vertical electrical sounding (VES) survey at the central part of
433 Kos island, Aegean, Greece. Geosciences 2006: 411–413.
434 Zouhri L, Gorini C, Mania J, Deffontaines B, Zeroulai A, Spatial distribution of resistivity in the
435 hydrogeological systems, and identification of the catchment area in the Rharb basin,
436 Morocco. Hydrological Sciences-Journal-des Sciences Hydrologiques, 2004; 49: 387-398.

[Click here to access/download;Figure;Fig. 1.jpg](https://www.editorialmanager.com/pone/download.aspx?id=32815620&guid=76b38d85-c8f5-4ab1-8cbb-ba8041ced8f2&scheme=1)

[Click here to access/download;Figure;Fig. 2.jpg](https://www.editorialmanager.com/pone/download.aspx?id=32815621&guid=6d1933f8-477b-49d5-910f-7934c1a35dc8&scheme=1)

[Click here to access/download;Figure;Fig. 3.jpg](https://www.editorialmanager.com/pone/download.aspx?id=32815622&guid=e8ade89d-0b55-4811-8f83-a5c697409875&scheme=1)

[Click here to access/download;Figure;Fig. 4.jpg](https://www.editorialmanager.com/pone/download.aspx?id=32815623&guid=ba401917-f528-4321-86c3-394552290a88&scheme=1)

[Click here to access/download;Figure;Fig. 5.jpg](https://www.editorialmanager.com/pone/download.aspx?id=32815624&guid=9760f011-fa13-490d-85e0-b526880b1f6b&scheme=1)

[Click here to access/download;Figure;Fig. 6.jpg](https://www.editorialmanager.com/pone/download.aspx?id=32815634&guid=f3f4c597-29d0-417f-8edb-bb37b880f618&scheme=1)

[Click here to access/download;Figure;Fig. 7.jpg](https://www.editorialmanager.com/pone/download.aspx?id=32815635&guid=e7d87763-8b75-4ee3-920d-38c56b33c490&scheme=1)

[Click here to access/download;Figure;Fig. 8.jpg](https://www.editorialmanager.com/pone/download.aspx?id=32815636&guid=f40edf14-c421-4e94-9ffe-8df474ce971a&scheme=1)

[Click here to access/download;Figure;Fig. 9.jpg](https://www.editorialmanager.com/pone/download.aspx?id=32815638&guid=466cc945-e848-4fa9-b430-3f5aefbab4d8&scheme=1)

[Click here to access/download;Figure;Fig. 10.jpg](https://www.editorialmanager.com/pone/download.aspx?id=32815639&guid=6ee04448-1a49-42f5-a1d4-fd1af0ac508d&scheme=1)
