# Supplementary material for: Hydrostratigraphy and hydrogeophysical studies to delineate fresh and saline aquifer boundaries in Lesser Cholistan of Pakistan
Source: PLoS One. 2023 Oct 5;18(10):e0292035. doi: 10.1371/journal.pone.0292035 (PMC10553205; doi:10.1371/journal.pone.0292035)
Supplement: Attachment — Submitted filename: Response to Reviewers.docx [file pone.0292035.s007.docx]

The authors are very thankful to the editor and the reviewers for their valuable time and comments. We have revised the manuscript in the light of the suggestions and comments of the reviewers and the editor. Please see the R1.docx. All important changes made in the manuscript are highlighted in RED COLOR text in the manuscript file.

The response of each comment is given below in tabular form. We are hopeful that the manuscript is now ready for acceptance.

Kind regards,

Perveiz Khalid (Ph.D.)

| **Sr. No.** | **Comments for Reviewer #1** | **Answers** |
| --- | --- | --- |
| 1 | Line 34: sources of freshwater (Hasan, 2017), It should be Hasan et al., 2017 | Done |
| 2 | Line#41: main source of groundwater contamination (Rasool, 2017), It should be Rasool et al., 2017 | Done |
| 3 | Line #47: dunes of low height (Farooq et al., 47 2001). Not included in the reference list | Replaced with (Ahmad 2011) which is included in the reference list |
| 4 | Line#58: Geyh and Ploethner, 2008. Please check the year. Not matched with the given reference list | Done. That is Geyh and Ploethner, 2015 |
| 5 | Line#66: Anwar and Bureste, 2011. Not included in the reference list | Now included in the reference list |
| 6 | Line# 68,69: Cited so many references without mentioning any particular findings. | Those references are representing the literature in which hydrogeological investigations with the help of some appropriate geophysical techniques and methods are used to assess the groundwater potential in confined or unconfined aquifer system. |
| 7 | Line #93 : Kemal et al., 1965. Not included in the reference list | Now corrected as “Kamal and Shamsi, 1965” |
| 8 | Please recheck the References, all reference list must be cited in the text and vice versa and should be written in the correct format | Done. all reference list is cited in the text and vice versa and is written in the correct format |
| 9 | Line 161-162  It is mentioned that ‘’ VES data is interpreted by using a combination of curve matching technique and computer iterative modeling to find out number of geo-electric layers in the study area” but nothing is mentioned about the curve matching technique. | Done. The curve matching technique is now explained briefly. |
| 10 | Line 168-172  For calibration and verification of VES results with borehole data, some of the VES points are acquired near to the existing boreholes/tubewells. Please explain briefly how you correlate lithology with true resistivity values. | Done. The calibration is now explained in manuscript. |
| 11 | All the VES data points were modeled (Fig.5) by using computer software IP2Win (IPI2WIN-1D computer program. 2000; Zananiri et al., 2006; Sultan et al., 2009; Akhter et al., 2012; Farid et al., 2013; Farid et al., 2014; Muhammad and Khalid, 2017). The cited references seem to be irrelevant , it is better to remove these references.  Line 175-177 | Irrelevant references are removed |
| 12 | In general calibration between lithology and 176 resistivities is established using available borehole information and resistivity data and is shown in 177 Table 1  Result and discussion Line 244-255:  Resistivity points (AH-38, YZ- 245 52, and YZ-58, KH-22, AH-40, and HS-22 – 25, YZ-68,69,70,71, and YZ-73) are discussed with reference to water quality but its location is not shown in Fig.1. It is suggested that VES, borehole and test hole should be labeled in Fig.1 | If we label all VES and borehole numbers in the Fig. 1 it will be very difficult to read all clearly and create massy data. However, we have added a new figure Fig. 6b to show the location of calibrated VES points and boreholes. Line 482 |
| 13 | There is no agreement of interpreted lithologies between Figs. 6,7,9 and 10 in the light of calibration given in Table 1. In addition, so many figures are presented in the manuscript but in conclusion, nothing is written/mentioned. Conclusions mostly consisting of theory and no quantitative results in conclusions. | Done. All the needful changes in Table 1 and Cross-section (Fig. 9) are completed and incorporated. Now Fig. 6,7,9, 10 are in accordance with each other.  Moreover, the results in conclusions are also modified accordingly. |
| 14 | In Fig.5, the depth of investigation (AB/2) of YZ-07 is 100 m and consists of 5 layers whereas in Fig.6 the interpreted is 200 m depth and consists of 4 layers.  Results and discussions are very poor. Scientific arguments are very poor in the discussion. Duplication is very common. | In Fig 6 it has 5 layers. 1^st^ layer has thickness of 1.5m therefore difficult to observe in Fig 6. YZ-07 depth of investigation is 120m, in Fig 6 5^th^ layer is extended upto 200m on the basis of modeled curve. |

| **Sr. No.** | **Comments for Reviewer #2** | **Answers** |
| --- | --- | --- |
| 1 | All the resolution of the figures should be improved. | Done. |
| 2 | Abstract should be revised as the authors should give brief introduction in 1 or 2 lines why they are doing this research? They may add a sentence that the present study is intended to identify hotspots for fresh water zones or ……….. | Done. Abstract is modified accordingly. |
| 3 | Line 121 – 123 An updated reference should be added to strengthen the statement. (Rainwater and the Sutlej River are the main recharge sources …………………………. The recharge rate can be divided into deep and shallow categories) | Updated reference is added (Geyh and Ploethner, 2015) |
| 4 | In the methodology section, there are many recent research studies available on groundwater, however the authors have added only one 2022 reference. It is good to add some fresh references in this section. | Done |
| 5 | What’s the time scale of the calibration and validation of model? | The time scale for calibration and validation of model with borehole and VES acquired data is from 2016 to 2022. |
| 6 | Finally, the author should give us recommendations, based on the results for future studies specifically for the study area. Do the authors recommend skimming wells for the area? If yes what should be the locations? The extraction of freshwater layers is used for which purpose? Agriculture? Drinking? Or any other use? | Done. Please see the Conclusion section. |

| **Sr. No.** | **Comments for Reviewer #3** | **Answers** |
| --- | --- | --- |
| 1 | I can't really follow the results and conclusions reached from the maps--for example I don't know where the rivers are or the paleochannels that control the resistivity values. | Done with needful changes. |
| 2 | I'm not clear how the author differentiates lithology from water salinity for some layers--for example how tell a clay from a sand containing saline water as both have low resistivity. | Done with needful changes. |
| 3 | How are the resistivity value cutoffs for each lithology/water salinity determined? It seems like you would need at least a couple wells with both lithology logs and resistivity logs so you could assign a resistivity value to a lithology | Done.  The cutoff resistivity values are defined generally by using literature and current VES results along with borehole data.  However, some requisite justifications are incorporated in the manuscript. |

| **Sr. No.** | **Comments for Reviewer #4** | **Answers** |
| --- | --- | --- |
| 1 | Line 60 Fig. 1: Base map of the study area showing VES and Borehole locations. Include the VES point numbers and the borehole numbers that you have mentioned in the paper. | If we label all VES and borehole numbers in the Fig. 1 it will be very difficult to read all clearly and create massy data. However, we have added a new figure Fig. 6.b to show the location of calibrated VES points and boreholes. Line 482 |
| 2 | Line 105 Kanker: Define what is kanker here? | Kankers are basically stiff pebbles usually made up of mud or silt. Explained in the manuscript. |
| 3 | Line 179 Table 1: Mentions the VES points and Boreholes used for calibrating the results given in the table. | Done. Please see Fig. 6b. Explained in the manuscript. |
| 4 | Line 220 - 223 : Statement requires clarity with proper referring to figure or citation. | Done. The figure No. is referred in text with proper explanation. |
| 5 | Line 263: Fig. 7a-h: Maps depicting the distribution of true resistivity across varying depths in two  264 dimensions. (the values in figures are not readable) | Figures have been revised. Please see Line |
| 6 | Line 272: (resistivity values between VES sites KH-22 and HS-25) these are not readable on figure. | Figure has been revised |
| 7 | Line 288: Fig. 8: Base map of the study area showing VES points, boreholes, and cross section AA’ (Label the VES points and borehole numbers also on this map. | If we label all VES and borehole numbers in the Fig. 1 it will be very difficult to read all clearly and create massy data. However, we have added a new figure Fig. 6.b to show the location of calibrated VES points and boreholes. Line 482 |
| 8 | Line 290: Fig. 9: a) Electrical resistivity distribution versus depth along cross section AA’, (vales along x axis are not readable). | Figure has been revised |
| 9 | Line 305: No clear boundary was marked or shown on the map only the fresh and saline water zones have been delineated , rephrase the statement accordingly. | Done. Rephrased accordingly. |

| **Sr. No.** | **Comments for Reviewer #5** | **Answers** |
| --- | --- | --- |
| 1 | Abstract is very simple and needs to modify as per standard. Include some results value. | Done. Abstract modified accordingly. |
| 2 | Collect irrigated water data from the irrigation department and correlates it with cultivated land through surface water. This data will provide more support to your study due to climate change issues, so it is necessary to educate the administration and people regarding water each drop's significance. Groundwater contamination is directly proportional to low water. | Few scattered patches of limited irrigated lands can be observed in the study area. Almost 70% of the study area is comprised of sand dunes with no irrigated land. Moreover, our analysis is for the exploration groundwater zones and fresh-saline water boundaries, so, it is not necessary to collect the irrigation data. |
| 3 | In line 62 where you wrote study area is part of the Upper Indus Basin, while another study showed Lesser Cholistan as part of the Lower Indus Basin (https://link.springer.com/article/10.1007/s00704-016-2007-3/figures/1) | Done. The word in this line is changed accordingly. |
| 4 | In the Introduction, use recent citations and write the significance of the geophysical study to the context of the Lesser Cholistan area. | Done. Added up more recent citations and significance of the study. |
| 5 | Clearly express and justified your objectives of the present study. Everyone knows Cholistan is a vast desert in Southern Punjab so its groundwater TDS would be high. You explain why this study was conducted and unique. | Done. Manuscript is edited accordingly. |
| 6 | Method is appropriate and clearly explained | Thank you for your compliment. |
| 7 | Result interpretation is better | Thank you for your compliment. |
| 8 | Conclusion needs some comparison among inside spatial data concerning depth and Hydrostratigraphy. Use results value under what outcome has been observed through the present study. | Done. The conclusion is modified accordingly. |
| 9 | Review and resolve English Grammatical issues. | Done. The whole manuscript is reviewed thoroughly and removed grammatical mistakes. |

| **Sr. No.** | **Comments for Reviewer #6** | **Answers** |
| --- | --- | --- |
| 1 | All Figs should be redrawn to make them clear | All Figures have been revised |
| 2 | please, list all VES models nearby boreholes in Fig 6. | We have added a new figure Fig. 6b to show the location of calibrated VES points and boreholes. Line 482 |
| 3 | All results are completely based on VES models, which do not accurate since true resistivity (shown by blue line in Fig 5) does not match with apparent resistivity data in most of models in Fig 5, such as Fig 5a, b, e and f. Please, redraw and correct. | The issue was in the resolution of figures. Therefore, the mentioned figures are replaced with high resolution ones. |
| 4 | Y axis scale in Fig 5 is not clear. Please, make y-axis clear like x-axis scale | Figure has been revised |
| 5 | Please simplify resistivity range in Table 1, i.e., only use less than for very low and greater than with very high | Done. Table 1 modified accordingly. |

| **Sr. No.** | **Comments for Reviewer #7** | **Answers** |
| --- | --- | --- |
| 1 | I appreciate the effort of the author/s that collected the huge geophysical data but need to elaborate more on the linkage between stratigraphy and hydrogeophysical study. | Thank you for your compliment. Moreover, the elaboration as per instruction is incorporated in manuscript in relevant places. |
| 2 | ‘The source of hazardous pollutants’ does not explain the type of pollutant found in the entire study area (line 49). | Done. Added up different pollutants in this line accordingly. |
| 3 | The statement mentioned in lines 99 and 100 is not matched the information illustrated in Fig 2. | Done. The statement in this line is reviewed accordingly. |
| 4 | Some text in the Figures is not clear and readable, especially in Fig 9. | All Figures have been revised |
| 5 | Fig 1 and 8 is repetition, the section may also be marked in Fig 1. | Fig.1 is the Base Map depicting the spatial location of study area, VES, boreholes. Fig. 8 is only for representing the line for cross section. However the information in both figures is same |
| 6 | The sequences of Figs numbers (7 and 8) mentioned in the text are not in order. | Done. The sequence is modified accordingly. |
| 7 | Can merge Fig 7 & 9 (because of almost the same information). | Fig. 7 show the different maps depicting true resistivity at different depth and Fig. 9 depict the cross section of line drawn in Fig. 8. |
| 8 | The borehole identification number must be mentioned. | Figure have been revised |
| 9 | Formatting (Line 369) | Done. |
| 10 | References found in the list but not cited in the text  Samson (Line 316)  Beaumont (Line 319)  Bhutta (Line 320)  Bowling (Line 323)  Burbank (Line 328)  Burbank (Line 331)  Burbank (Line 334)  Curell (Line 341)  Geyh 2015 (Line 354)  Kamal (Line 361)  Khalid (Line 363)  Muhammad (Line 374)  Pakistan water partnership (Line 385)  Qureshi (Line 387)  Qureshi (Line 390)  Rasool (Line 393)  Sultan 2014 (Line 411)  Yang (Line 426)  Yu (Line 429). | Done. All references are adjusted after thorough review. |
| 11 | References cited in the text but not found in the list  Rasool (Line 41)  Farooq (Line 46)  Geyh and Ploethner 2008 (Line 58)  Farooq (Line 63)  Anwar and Bureste (Line 66)  Farid (Line 80)  Kemal (Line 93)  Farooq (Line 134)  Louis (line 145)  IPI2WIN-1D computer program. 2000 (lines 170-171)  Sultan (line 171)  Akhter (line 171)  Muhammad and Khalid (line 172) | Done. All references are adjusted after thorough review. |
